# Novel 1,2,3-Triazole-Containing Quinoline–Benzimidazole Hybrids: Synthesis, Antiproliferative Activity, In Silico ADME Predictions, and Docking

**DOI:** 10.3390/molecules28196950

**Published:** 2023-10-06

**Authors:** Luka Krstulović, Katarina Mišković Špoljarić, Vesna Rastija, Nikolina Filipović, Miroslav Bajić, Ljubica Glavaš-Obrovac

**Affiliations:** 1 Department of Chemistry and Biochemistry, Faculty of Veterinary Medicine, University of Zagreb, Heinzelova 55, 10000 Zagreb, Croatia; mbajic@vef.hr; 2 Department of Medicinal Chemistry, Biochemistry and Clinical Chemistry, Faculty of Medicine, Josip Juraj Strossmayer University of Osijek, Josipa Huttlera 4, 31000 Osijek, Croatia; kmiskovic@mefos.hr; 3 Faculty of Agrobiotechnical Sciences Osijek, Josip Juraj Strossmayer University of Osijek, Vladimira Preloga 1, 31000 Osijek, Croatia; vrastija@fazos.hr; 4 Department of Chemistry, Josip Juraj Strossmayer University of Osijek, Cara Hadrijana 8a, 31000 Osijek, Croatia; nfilipovic@kemija.unios.hr

**Keywords:** 1,2,3-triazole-containing quinoline–benzimidazole hybrids, synthesis, antiproliferative effect, in silico ADME, docking

## Abstract

The newly synthesized quinoline–benzimidazole hybrids containing two types of triazole-methyl-phenoxy linkers were characterized via NMR and elemental analysis. Additional derivatization was achieved by introducing bromine at the C-2 position of the phenoxy core. These novel hybrids were tested for their effects on the growth of the non-tumor cell line MRC-5 (human fetal lung fibroblasts), leukemia and lymphoma cell lines: Hut78, THP-1 and HL-60, and carcinoma cell lines: HeLa and CaCo-2. The results obtained, presented as the concentration that achieves 50% inhibition of cell growth (IC_50_ value), show that the compounds tested affect tumor cell growth differently depending on the cell line and the dose applied (IC_50_ ranged from 0.2 to >100 µM). The quinoline–benzimidazole hybrids tested, including 7-chloro-4-(4-{[4-(5-methoxy-1H-1,3-benzo[d]imidazol-2-yl)phenoxy]methyl}-1H-1,2,3-triazol-1-yl)quinoline **9c**, 2-(3-bromo-4-{[1-(7-chloroquinolin-4-yl)-1H-1,2,3-triazol-4-yl]methoxy}phenyl)-N-propyl-1H-benzo[d]imidazol-5-carboximidamide trihydrochloride **10e**, 2-{4-[(1-{2-[(7-chloroquinolin-4-yl)amino]ethyl}-1H-1,2,3-triazol-4-yl)methoxy]phenyl}-N-propyl-1H-benzo[d]imidazol-5-carboximidamide trihydrochloride **14e** and 2-{3-bromo-4-[(1-{2-[(7-chloroquinolin-4-yl)amino]ethyl}-1H-1,2,3-triazol-4-yl)methoxy]phenyl}-N-propyl-1H-benzo[d]imidazol-5-carboximidamide trihydrochloride **15e**, arrested the cell cycle of lymphoma (HuT78) cells. The calculated ADMET properties showed that the synthesized compounds violated at most two of Lipinski’s rules, making them potential drug candidates, but mainly for parenteral use due to low gastrointestinal absorption. The quinoline–benzimidazole hybrid **14e**, which was shown to be a potent and selective inhibitor of lymphoma cell line growth, obtained the highest binding energy (−140.44 kcal/mol), by docking to the TAO2 kinase domain (PDB: 2GCD).

## 1. Introduction

Nitrogen-containing heterocycles are ubiquitous in nature and represent the most common scaffold in approved drugs. An analysis of small molecule therapeutics approved by the FDA in the last five years shows that more than half of the approved drugs contain aromatic *N*-heterocycles [1,2,3,4,5]. *N*-heterocycles are also a component of numerous small molecules that have been approved by the FDA for the treatment of cancer in recent years [6].

Cancer is one of the leading causes of death worldwide, with an estimated 10 million deaths in 2020 [7,8]. Despite tremendous efforts, drug resistance is still one of the main reasons for cancer treatment failure [9,10]. One of the latest strategies for cancer therapy is the use of molecular hybrids. The goal of molecular hybridization is the covalent binding of two or more pharmacophores in a single molecule that has the ability to eliminate drug resistance and improve selectivity [11,12,13]. We aimed to apply this approach to the preparation of hybrids containing quinolines linked to benzimidazoles via a 1,4-disubstituted 1,2,3-triazole linker. To this end, we used a well-known, robust, reliable, and efficient copper-catalyzed cycloaddition of azides and terminal alkynes [14].

Click chemistry has made compounds containing 1,2,3-triazole readily available. Due to its ability to act as a linker, pharmacophore and bioisoster, the 1,2,3-triazole motif is of great importance in drug discovery and can be found in the structures of drug candidates and approved drugs [15,16,17].

**Figure 1:**
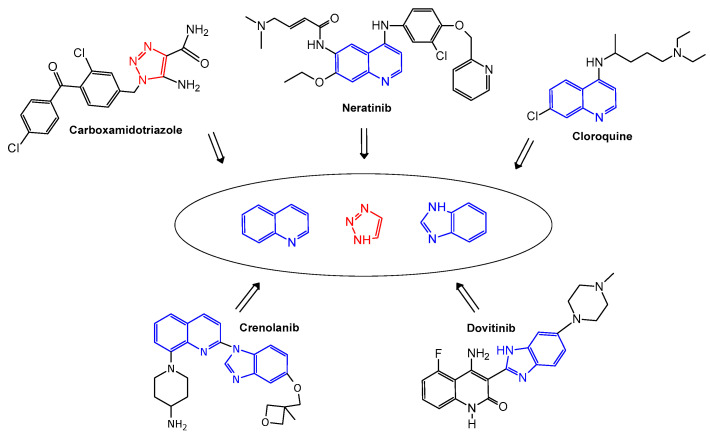
Design of novel triazole-containing benzimidazole–quinoline hybrids.

Over the years, numerous triazole compounds have also been tested as potential anticancer drugs [17,18,19,20]. Carboxyamidotriazole, shown in Figure 1, is a calcium channel blocker with a 1,4,5-disubstituted 1,2,3-triazole backbone. Recently, carboxyamidotriazole has been clinically investigated as an orotate prodrug salt in non-small cell lung cancer, glioblastoma, and anaplastic glioma [21,22].

Quinoline is one of the preferred scaffolds in drug discovery and there are numerous reports on the antiviral, antibacterial, antifungal, antiviral, and antiparasitic activities of compounds with quinoline structure [23,24]. For years, quinoline has played an important role in the discovery of anti-cancer agents [25,26]. The 4-amino-7-chloroquinoline-based antimalarials chloroquine and hydroxycloroquine have been studied in more than sixty clinical trials as anticancer therapies (Figure 1) [27]. Neratinib, shown in Figure 1, is a quinoline-based therapeutic that acts as a tyrosine kinase inhibitor and is used to treat the early and advanced stages of HER-2 positive breast cancer. It has also been clinically evaluated for the treatment of patients with HER-2 mutated advanced bile duct cancer [28,29,30]. Benzimidazole, also known as 1*H*-benzimidazole and 1,3-benzodiazole, is an aromatic bicyclic *N*-heterocycle containing a benzene ring fused to an imidazole ring. Benzimidazole-containing compounds have shown a wide range of biological activities and have been attracting attention in the drug discovery field for years [31,32]. The benzimidazole backbone is present in several drugs approved for cancer therapy [33]. A patent survey of benzimidazoles shows that more than half of the new benzimidazole compounds were developed for cancer treatment during the indicated period [34]. Crenolanib (Figure 1) is in clinical trials for acute myeloid leukemia and acts as an FMS-like tyrosine kinase-3 inhibitor. Structurally, it is a compound containing a benzimidazole directly linked to a quinoline moiety [35,36]. Dovitinib is also an anticancer agent containing a benzimidazole scaffold directly linked to a quinolinone. It is a tyrosine kinase inhibitor and has been used in clinical trials to treat patients with advanced pancreatic cancer [37].

Considering the antiproliferative activity of compounds with the above components and continuing our work on quinoline–benzimidazole hybrids [38,39], we report here our next step in the search for anticancer agents, which involves the synthesis of triazole-linked quinoline–benzimidazole hybrids and the evaluation of their antiproliferative activity. To elucidate the possible mechanisms of the antiproliferative activities of the investigated compounds, we performed a molecular docking study on TAO2 (thousand-and-one amino acids) protein kinase. In addition, in silico physicochemical and pharmacokinetic/ADMETstudies (absorption, distribution, metabolism, excretion, and toxicity) were performed to theoretically predict their behavior as drug candidates.

## 2. Results and Discussion

### 2.1. Chemistry

**Scheme 1:**
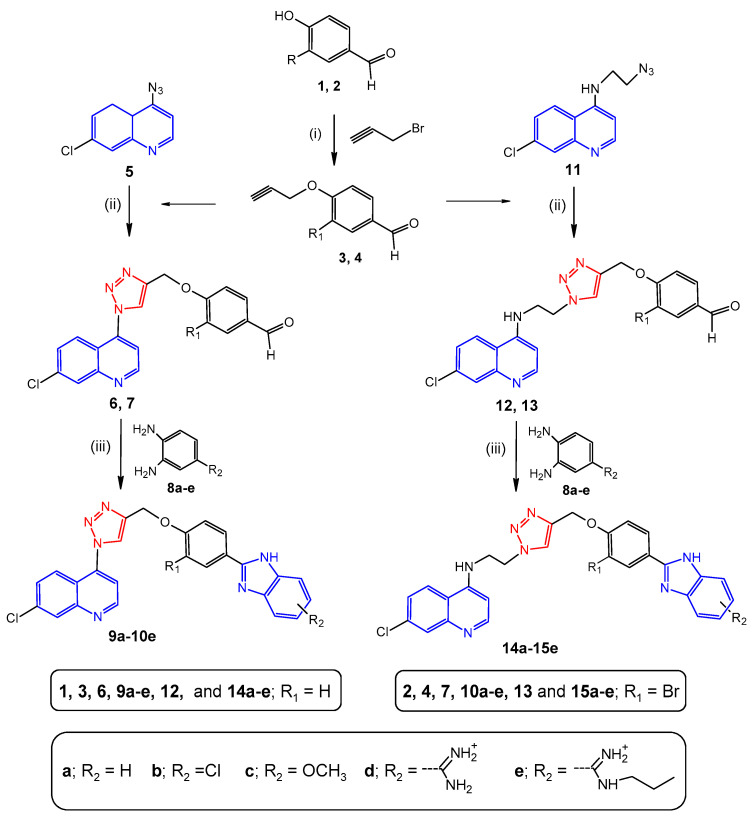
Reagents and reaction conditions: (i) K_2_CO_3_, DMF, 30 °C, 24 h; (ii) sodium ascorbate, CuSO_4_, DMF, 65 °C, 24 h; (iii) Na_2_S_2_O_5_, DMSO, 165 °C, 15 min.

The synthesis of the new quinoline–benzimidazole compounds **9a**–**10e** and **14a**–**15d** was carried out as shown in Scheme 1. We prepared hybrid molecules containing quinoline and benzimidazole units linked to two types of triazole methyl phenoxy linkers. The target compounds bear two non-amidine and two amidine substituents at the C-5 position of the benzimidazole molecule. We attempted additional derivatization with a bromine substituent at the C-2 position of the phenoxy core.

The required intermediates **3**, **4**, **5**, and **11** as well as benzene-1,2-diamines **8d**–**e** were prepared according to the previously reported procedures [40,41,42].

Triazole intermediates **6**, **7**, **12**, and **13** were synthesized using a partially modified Cu(I)-catalyzed azide-alkyne cycloaddition previously reported by Guntai et al. [40]. Target compounds **9a**–**10e** and **14b**–**15d** were prepared using triazole intermediates and the corresponding benzene-1,2-diamines **8a**–**e** in the presence of sodium metabisulfite in DMSO at 165 °C [43].

### 2.2. Biological Activity

#### 2.2.1. Evaluation of Antiproliferative Activity of the Novel Compounds

The newly synthesized 1,2,3-triazole-containing quinoline–benzimidazole hybrids were tested to determine their effect on cell growth. The cell lines selected for evaluation included a range of non-tumor and tumor cells: MRC-5 (human fetal lung fibroblasts, non-tumor), Hut78 (T-cell lymphoma), THP-1 (acute monocytic leukemia), HL-60 (acute promyelocytic leukemia), HeLa (human cervical adenocarcinoma), and CaCo-2 (adenocarcinoma of colon). The results of the experiments, quantified according to the concentration at which 50% growth inhibition (IC_50_ value) was achieved, showed different effects of the studied compounds on tumor cell growth. The results depended on factors such as the cell line tested and the dose administered. This suggests that the effects of the compounds on cell growth are influenced by a complex interplay of factors, including their chemical structure, cellular context, and dose-dependent effects.

**Figure 2:**
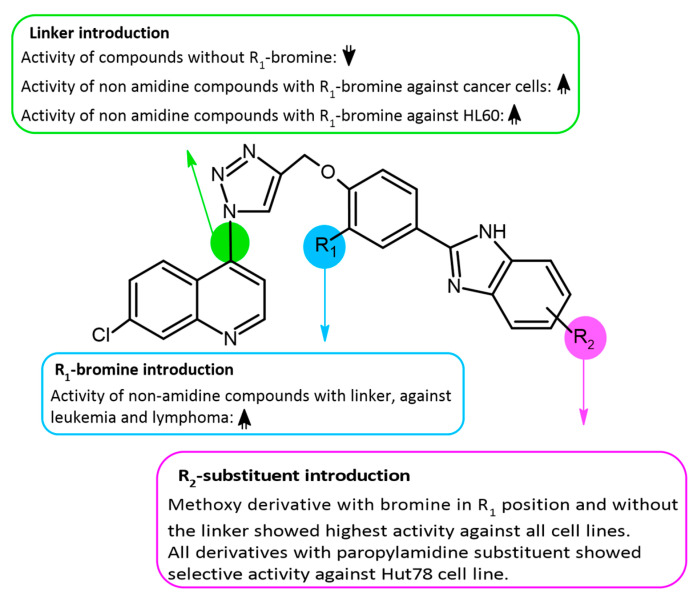
Structure activity relationship for antiproliferative activity of the 1,2,3-triazole-containing quinoline–benzimidazole hybrids.

As shown in Table 1, the IC_50_ values ranged from 0.18 to over 100 µM for all compounds tested. Carcinoma cell lines were more resistant to the tested compounds compared to leukemia and lymphoma cells. In the group of non-amidine compounds without ethylamino linker **(9a**–**9c)**, it was observed that compound **9c** with the methoxy group on the benzimidazole moiety (R_2_) exhibited the highest cytotoxicity and showed a non-selective effect against tumor cells compared to normal cells (Figure 2). Although **9c** showed a significant effect on the growth of normal MTR-5 cells with a IC_50_ of 2.71 µM, its selectivity index (SI) was 15 for THP cells (IC_50_: 0.18 µM) and 13.55 for HuT78 cells (IC_50_: 0.18 µM). Amidine compounds **9d** and **9e**, containing amidine and propylamidine substituents on the benzimidazole core, respectively, caused a selective and pronounced antiproliferative effect on HuT78 cells (**9d**: IC_50_ = 2.52 µM; SI = 39.7; **9e**: IC_50_ = 10.2 µM; SI = 9.8).

In the group of compounds without ethylamino linker and with added bromine at the phenoxy core (**10a**-**e**), the non-amidine compounds **10a** and **10b** showed increased toxicity against both normal and tumor cell lines. The replacement of hydrogen with chlorine at the benzimidazole in **10b** increased antiproliferative activity in all tumor cell lines tested compared with the effect of **9b**. Compound **10c**, which has a methoxy group on the benzimidazole, showed lower cytotoxic activity than compound **9c**, which lacked the bromine substitution at the R_1_ position. Compound **10d** (with an amidine group at the R_2_ position) showed insignificant inhibitory potential against normal and tumor cells, while derivative **10e** (with a propylamidine group at the R_2_ position) showed selective activity against HuT78 cells in terms of growth of normal cells (IC_50_ = 9.68 µM; SI = 9.8).

In the group of quinoline–benzimidazole hybrids bearing an ethylamino linker (**14a**–**e**), the non-amidine compounds **14a** and **14c** showed no inhibitory effect on the tested cell lines, except for **14c**, which showed selective activity against THP-1 cells (IC_50_ = 7.31 µM; SI = 13.7). Interestingly, the hybrids with amidine or propilamidine at the C-5 position of the benzimidazole moiety, **14d** and **14e**, showed no growth inhibition in the tested cell lines and showed almost identical selective antiproliferative activity only in HuT78 cells (**14d**: IC_50_ = 10.51 µM; SI = 9.51; **9e**: IC_50_ = 10.86 µM; SI = 9.2).

The introduction of bromine at the phenoxy core in the group of quinoline–benzimidazole hybrids containing an ethylamino linker (**15a**–**e)** significantly alters the inhibitory effects of non-amidine compounds **15a**–**c** on the growth of leukemia and lymphoma cells compared to non-substituted non-amidine compounds **14a**–**c** (Table 1 and Figure 2). Compared with MRC-5 cells and carcinoma cell lines, **15a**–**c** showed significant antiproliferative activity against lymphoma cells HuT78 (IC_50_ ranged from 3.98 to 7.88 µM and leukemia cells (HL-60: IC_50_ ranged from 1.21 to 11.63 µM; THP1: IC_50_ ranged from 1.65 and 4.66 µM).

No significant difference in the antiproliferative effect was observed between the normal MRC-5 and carcinoma cell lines (HeLa and CaCo2) for compounds **15a**–**c** (Table 1). The introduction of amidine to benzimidazole (**15d**) resulted in a loss of antiproliferative activity, whereas the introduction of propilamidine to benzimidazole (**15e**) resulted in selective activity against HuT78 cells (IC_50_ = 7.83 µM; SI = 12.8).

#### 2.2.2. Cell Cycle Perturbation

**Figure 3:**
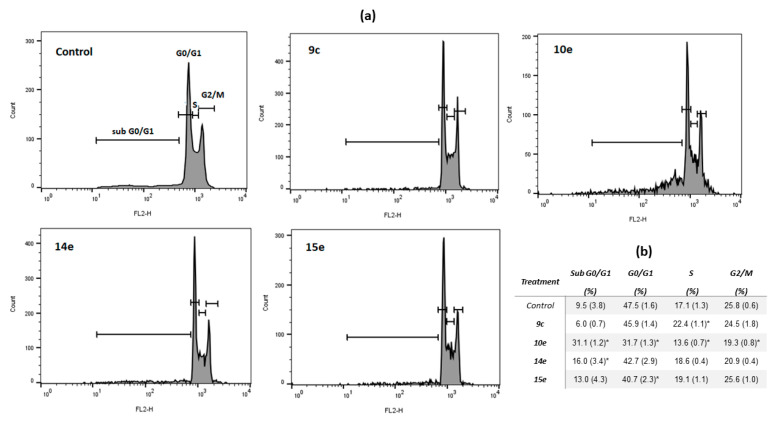
Flow cytometry analysis of the cell cycle distribution of HuT-78 cells exposed to compounds **9c** (0.5 µM) and **10e**, **14e** and **15e** (5 µM) for 24 h. (**a**) DNA histograms present changes in cell cycle. (**b**) Data are expressed as mean ± standard deviation. A statistically significant *p* value is defined as *p* < 0.05 (*).

Inducing cell cycle arrest at specific checkpoints and inducing apoptosis are common strategies in cancer treatment with cytotoxic agents. Numerous studies have shown that quinoline- and benzimidazole-based compounds, as well as their hybrids, significantly affect tumor cell growth [44,45]. To determine whether cell cycle arrest underscores the antiproliferative activity that some compounds exhibit at micromolar and submicromolar concentrations against lymphoma and leukemia cells, we tested the cell cycle distribution of HuT-78 treated with hybrids containing amidine or propylamidine at the C-5 position of the benzimidazole moiety (**10e**, **14e** and **15e**), which showed a selective effect on HuT78 cells growth. The effect on the cell cycle of the non-amidine compound without an ethylamino linker, compound **9c**, which has a methoxy group on the benzimidazole core (R_2_) and showed submicromolar IC_5_ on leukemia and lymphoma cells (Table 1), were also investigated. As shown in Figure 3, compound **9c** caused a statistically significant enrichment of the S fraction (at 31%, *p* < 0.05) and a significant decrease in the G1 phase (at 66.7%, *p* < 0.05) compared to control cells. No significant changes were observed in the other phases of cell cycle. In contrast to compound **9c**, compound **10e** with introduced bromine at the phenoxy core and with a propylamidine group at the R_2_ position (without an ethylamino linker) caused a statistically significant increase in the aggregation of cells in the subG0/G1 phase (more than threefold, *p* < 0.05) with a corresponding decrease in the G1, S and G2/M phases of the cell cycle. In addition, the results of the test of the effects of quinoline–benzimidazole hybrids containing an ethylamino linker showed that the compound with propylamidine at the C-5 position of the benzimidazole moiety, **14e**, and the compound with bromide at the phenoxy core, **15e**, similarly affected the cell cycle by increasing the proportion of cells in subG0/G1 phase (**14e**: by 68%; **15e**: by 36%, *p* < 0.05), resulting in a reduction in the number of cells in G1 and G2/M phases compared to untreated cells, while the changes in other phases of the cell cycle were not statistically significant. These results suggest that the entry of treated cells into a new cell cycle is prevented and are consistent with the results published by Zuo at al. and Zhang at al. [46,47]. The subG0/G1 arrest suggests that the initiation of cell cycle arrest may be responsible for the antiproliferative potential. The subG0/G1 peak indicates DNA fragmentation and suggests that the cells die, most likely through apoptosis. The arrest of the cell cycle in the G2/M phase in cells treated with various benzimidazole derivatives has also been observed in several recently published studies [45]. These compounds likely affect different phases of the cell cycle, resulting in changes in cell proliferation and growth.

### 2.3. Absorption, Distribution, Metabolism, Excretion (ADME), and Toxicity Properties

Calculated ADME, pharmacokinetic and druglike properties of the analyzed compounds were presented in Table 2. According to the “Lipinski’s rule of 5”, the physicochemical ranges for high probability to be an oral drug are molecular weight (MW), between 150 and 500 g/mol; saturation fraction of carbons in the sp^3^ hybridization, not less than 0.25; flexibility: rotatable bonds < 9; lipophilicity, XLOGP3 between −0.7 and +5.0; polarity, topological polar surface area (TPSA) between 20 and 130 Å^2^; water solubility, log S < 6 [48].

**Figure 4:**
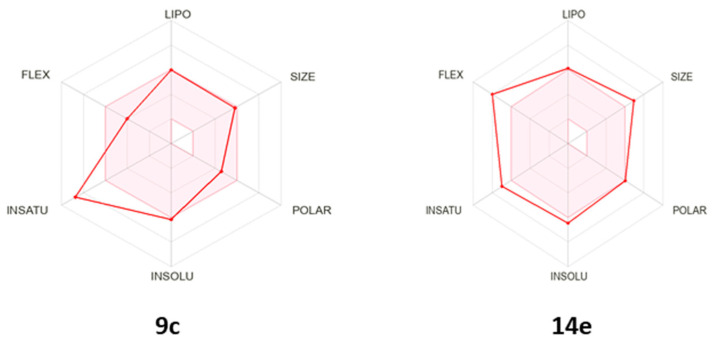
Bioavailability radars for the two most active compounds **9c** and **14e**. The pink area represents the optimal range for each property (lipophilicity (XLOGP3); size (MW); polarity (TPSA); water solubility (log S); saturation (Fraction Csp3); and flexibility (number of rotatable bonds, FLEX).

The drug similarity of all 20 quinoline–benzimidazole hybrids is schematically represented by the bioavailability radars in the Supplementary Materials (Table S1), while that for the selected molecules is presented in Figure 4.

Six physicochemical properties are taken into account: lipophilicity, size, polarity, solubility, flexibility and saturation [49]. Compound **9c** displayed antiproliferative affinities against all tested tumor cell lines, especially on Hut78 cells (IC_50_ = 0.2 µM) (Table 1). It also exhibited a toxic effect on normal MTR-5 cells but with great selectivity toward lymphoma cell line Hut78. That compound does not violate either one of the Lipinski rules, making it an ideal drug candidate. According to its bioavailability radar, compound **9c** has optimal range of all properties, except for the fact that it is low in saturation index. It means that the ratio of sp^3^ hybridized carbons over the total carbon count of the molecule (fraction Csp^3^) is lower than 0.25. Compound **9c** is poorly soluble in water, but it has high gastrointestinal absorption (GIA), which is important for effective oral drugs. It also has low blood–brain barrier permeation (BBBP), which means that is safe for the unwanted effects of peripheral drugs on the brain (Table 2) [50]. The ability to be transported by P-glycoprotein (P-gp) out of the cell is an important ADME property of compound **9c**. It means that it could be eliminated from cells, thereby preventing intracellular accumulation and decreasing toxicity. Compound **14e** showed strong selective activity against HuT78 cells (IC_50_ = 10.86 µM) (Table 1). It breaks only one Lipinski rule since it is MW > 500 g/mol (Table 2). The bioavailability radar of compound **14e** reveals its inappropriate drug properties: large size (MW > 500 g/mol); low in saturation index (fraction Csp^3^ = 0.19); flexibility (number of rotatable bonds > 9). Since that compound is poorly soluble in water, has a low GIA index, and cannot act as a P-glycoprotein substrate, it could only be used for parenteral drug administration.

### 2.4. Molecular Docking Study

**Figure 5:**
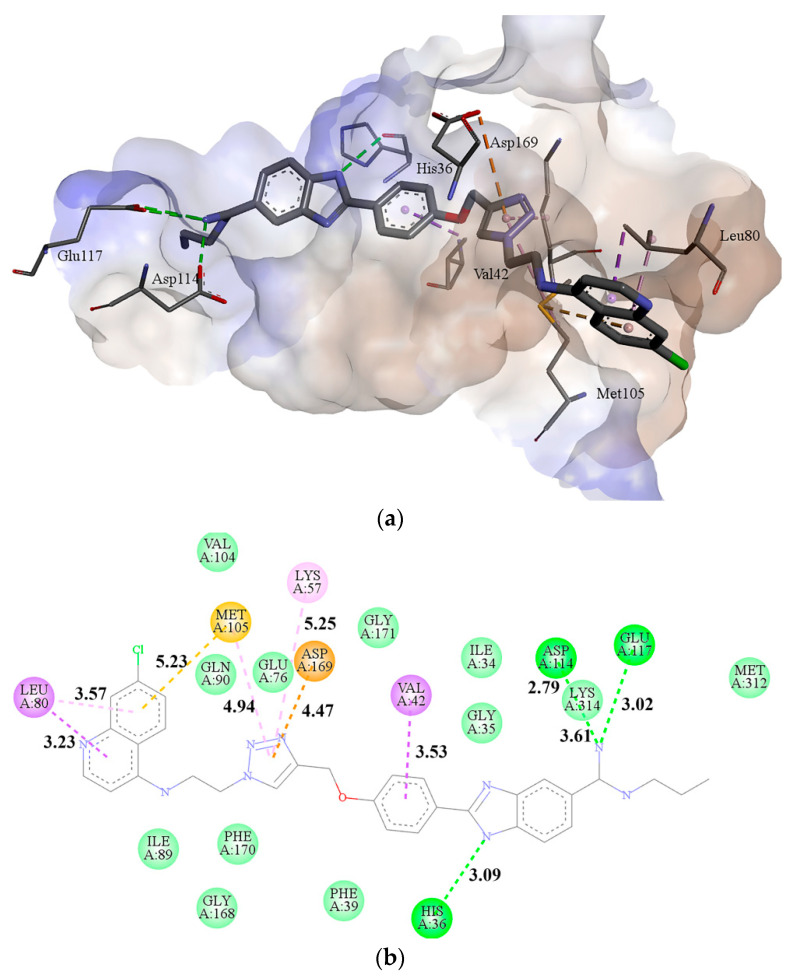
(**a**) Hydrophobic surface representation of the ATP-binding cleft of TAO2 with docked compound **14e**. (Hydrophobicity range: brown = 3; white = 0; blue = −3); (**b**) 2D representation of the main interactions between compound **14e** and residues of the TAO2 kinase with the showed distances (Å). (green = conventional hydrogen bond; light green = van der Waals interactions; brown = π-anion; purple = π–σ interactions; pink = π–alkyl interactions).

Molecular docking of 20 quinoline–benzimidazole hybrids was performed on the binding site of the TAO2 kinase domain defined according to the bonded ligand staurosporine (STU) (PDB: 2GCD). STU is a well-known inducer of apoptosis in a wide range of the tumor cell lines. The crystal structure of this complex was chosen because it provides the details of the interactions between TAO2and staurosporine, which explains the relatively low potency of staurosporine against TAO2. Although STU is too toxic to be used directly as a therapeutic agent, it could serve as a template for the design of inhibitors specific to TAO2 [51]. Compounds were ranked by the total energy of the predicted pose in the binding site (Table 3), and the docking scores were compared with the docking results of the staurosporine. The highest binding energy on the TAO2 kinase domain, even before standard ligand staurosporine, was observed for compound **14e** (−140.44 kcal/mol), which has also exhibited a strong selective antiproliferative effect on HuT78 cells (IC_50_ = 10.86 µM). The docking result of STU is followed by strong selective inhibitors of HuT78 cells: **9e**, **14d**, **9d**, and **10e**. The energies of the main interactions between compound **14e** and residues in the binding site of the TAO2 kinase are shown in Table 4. The position of this compound into the binding site of the TAO2 kinase domain represented as a hydrophobic surface is shown in Figure 5a, while its interactions with amino acid residues are presented using a 2D diagram (Figure 5b).

Compound **14e** is located in the hydrophobic surface of the ATP-binding cleft of the TAO2 due to the presence of plenty of hydrogen bonds and van der Waal interactions. Compound **14e** creates major hydrogen bonds through the N atom of benzimidazole scaffold with His36 (3.09 Å), and the N atom of propilamidine moiety with Asp114 (2.79 Å) and Glu117 (3.02 Å). Van der Waals interactions are very similar to those observed in staurosporine complex with TAO2 kinase (PDB: 2GCD): Gly35; Ile34; Phe39; Glu76; Gln90; Ile89; Gly168; Phe170; Met312. The other interactions are as follows: π–σ interactions (Leu80; Val42); π-anion (Met105; Asp169); π–alkyl interactions (Lys57). We note the very tight π–σ interaction of the quinolone moiety with Leu80 (3.23 Å), leading to a strong interaction energy (11.43 kcal/mol). The interactions between TAO2 and staurosporine involve three hydrogen bonds with Glu106, Cys108, and Gly155 [51].

Mitogen-activated protein kinases (MAPKs) are involved in signal transduction pathways in eukaryotic cells, controlling multiple cellular programs. MAPKs are activated by MAPK kinases. TAO2 is a MAP3K level kinase that activates p38 MAPKs, which regulate the production of cytokines. TAO2 is activated in response to apoptosis-inducing agents and acts as regulators of apoptosis; it represents a potential drug target for the treatment of cancer [52]. TAO2 is thought to be a physiological regulator of p38 because it is activated during the differentiation of C2C12 myoblasts in parallel with activation of p38 [53]. Therefore, TAO2 is a potential target for the treatment of p38 MAPK-associated diseases such as leukemia, breast cancer, prostate cancer, bladder cancer, liver cancer, lung cancer, thyroid cancer, and many others. Selective inhibitors of TAO2, which inhibit the regulation of MAPK signaling pathway, are used for the development of potential cancer therapy [54].

## 3. Materials and Methods

### 3.1. Chemistry

All solvents and reagents were used without purification from commercial sources. To monitor the progress of a reaction and for comparison purposes, thin layer chromatography (TLC) was performed on precoated Merck silica gel 60F-254 plates using an appropriate solvent system, and the spots were detected under UV light (254 nm). Melting points (uncorrected) were determined using the Buchi 510 melting point instrument. ^1^H and ^13^C NMR spectra were recorded using a Bruker Avance DPX-300 or Bruker AV-600 spectrometer. All data were recorded in DMSO-d6 at 298 K. Chemical shifts were related to the residual solvent signal of DMSO at d 2.50 ppm for ^1^H and d 39.50 ppm for ^13^C. Elemental analyses for carbon, hydrogen and nitrogen were performed using the Perkin-Elmer 2400 elemental analyzer. The analyses are given as symbols of the elements. The analytical results obtained are within 0.4% of the theoretical value.

#### 3.1.1. General Procedure for the Synthesis of Compounds **6**, **7**, **12** and **13**

Aldehyde **3** or **4** (1 mmol) and the appropriate azide **5** or **11** (1 mmol) were dissolved in 5 mL DMF and, while stirring at 65 °C, 0.4 mL of 1M sodium ascorbate water solution and 0.2 mL of 1M CuSO_4_ water solution were added. The reaction mixture was then stirred at 65 °C for 24 h. The reaction mixture was filtered through a short Al2O3 column, with the addition of water, and the product was precipitated.

##### 4-((1-(7-chloroquinolin-4-yl)-1H-1,2,3-triazol-4-yl)methoxy)benzaldehyde (**6**)

Compound **6** was prepared using the above-described method from **3** (261 mg, 1.5 mmol), **5** (307 mg, 1.5 mmol), 0.6 mL of 1M sodium ascorbate water solution and 0.3 mL of 1M CuSO_4_ water solution, as off white product (416 mg, 77%); mp = 188–190 °C. ^1^H NMR (600 MHz, DMSO-*d*6) δ/ppm 9.91 (s, 1H, CHO), 9.17 (d, *J* = 4.6 Hz, 1H, ArH), 9.03 (s, 1H, triaz.), 8.30 (d, *J* = 1.9 Hz, 1H, ArH), 8.00 (d, *J* = 9.1 Hz, 1H, ArH), 7.92 (d, *J* = 8.8 Hz, 2H, ArH), 7.89 (d, *J* = 4.6 Hz, 1H, ArH), 7.80 (dd, *J* = 2.0 Hz, *J* = 9.1 Hz, 1H, ArH), 7.33 (d, *J* = 8.8 Hz, 2H, ArH), 5.47 (s, 2H, CH_2_). ^13^C NMR (151 MHz, DMSO-*d*6) δ/ppm 191.33, 162.83, 152.33, 149.34, 142.97, 140.27, 135.37, 131.80, 129.99, 128.97, 127.53, 125.31, 120.26, 117.14, 115.24, 61.20. Anal. calcd. for C_19_H_13_ClN_4_O_2_ × H_2_O (*M*r = 382.80): C 59.61, H 3.95, N 14.64; found: C 59.68, H 4.19, N 14.32.

##### 3-bromo-4-{[1-(7-chloroquinolin-4-yl)-1H-1,2,3-triazol-4-yl]methoxy}benzaldehyde (**7**)

Compound **7** was prepared using the above-described method from **4** (1.08 g, 4.9 mmol), **5** (1 g, 4.9 mmol), 2 mL of 1M sodium ascorbate water solution and 1 mL of 1M CuSO_4_ water solution, as an off-white product (1.6 g, 74%); mp = 163–165 °C. ^1^H NMR (600 MHz, DMSO-*d*6) δ/ppm 9.88 (s, 1H, CHO), 9.17 (d, *J* = 4.6 Hz, 1H, ArH), 9.03 (s, 1H, triaz.), 8.31 (d, *J* = 2.1 Hz, 1H, ArH), 8.14 (d, *J* = 1.9 Hz, 1H, ArH), 7.99 (m, 2H, ArH), 7.90 (d, *J* = 4.6 Hz, 1H, ArH), 7.81 (dd, *J* = 2.1 Hz, *J* = 9.0 Hz, 1H, ArH), 7.65 (d, *J* = 8.6 Hz, 1H, ArH), 5.58 (s, 2H, CH_2_). 13C NMR (151 MHz, DMSO-*d*6) δ/ppm 190.54, 158.82, 152.30, 149.34, 142.59, 140.20, 142.59, 135.35, 134.06, 131.05, 130.92, 128.98, 127.26, 125.27, 120.22, 117.14, 114.16, 111.78, 62.32. Anal. calcd. for C_19_H_12_ClBrN_4_O_2_ × 0.5H_2_O (*M*r = 452.69): C 50.41, H 2.89, N, 12.38; found: C 50.59, H 2.78, N 12.54.

##### 4-[(1-{2-[(7-chloroquinolin-4-yl)amino]ethyl}-1H-1,2,3-triazol-4-yl)methoxy]benzaldehyde (**12**)

Compound **12** was prepared using the above-described method from **3** (364 mg, 2.1 mmol), **11** (515 mg, 2.1 mmol), 0.84 mL of 1M sodium ascorbate water solution and 0.42 mL of 1M CuSO_4_ water solution, as a beige product (648 mg, 76%); mp = 121–123 °C. ^1^H NMR (600 MHz, DMSO-*d*6) δ/ppm 9.87 (s, 1H, CHO), 8.28 (brs+s, 2H, ArH), 7.85 (brs+d, *J* = 8.6 Hz, 3H, ArH), 7.48 (s, 1H, NH), 7.45 (d, *J* = 8.9 Hz, 1H, ArH), 7.20 (d, *J* = 8.6 Hz, 2H, ArH), 6.75 (s, 1H, ArH), 5.25 (s, 2H, CH_2_), 4.68 (t, *J* = 5.8 Hz, 2H, CH_2_), 3.78 (m, 2H, CH_2_). ^13^C NMR (151 MHz, DMSO-*d*6) 191.26, 162.89, 141.95, 133.18, 131.71, 129.79, 125.31, 124.52, 115.12, 61.41, 47.81, 42.42, 35.79. δ/ppm 190.84. Anal. calcd. for C_21_H_18_ClN_5_O_2_ × 2H_2_O (*M*r = 443.88): C 56.82, H 5.00, N, 15.78; found: C 56.65, H 5.23, N 15.96.

##### 3-bromo-4-[(1-{2-[(7-chloroquinolin-4-yl)amino]ethyl}-1H-1,2,3-triazol-4-yl)methoxy]benzaldehyde (**13**)

Compound **13** was prepared using the above-described method from **4** (400 mg, 1.8 mmol), **11** (442 mg, 1.8 mmol), 0.72 mL of 1M sodium ascorbate water solution and 0.36 mL of 1M CuSO_4_ water solution, as a beige product (530 mg, 31%); mp = 125–127 °C. ^1^H NMR (600 MHz, DMSO-*d*6) δ/ppm 9.84 (s, 1H, CHO), 8.75–8.15 (brs+s, 3H, ArH), 8.08 (d, *J* = 1.3 Hz, 1H, ArH), 7.88 (d, 7.48 (s, *J* = 8.5 Hz 1H, ArH), 7.62–7.19 (brs+d+d, *J* = 8.8 Hz, *J* = 8.5 Hz, 4H, NH+ArH), 6.95 (brs, 1H, ArH), 5.36 (s, 2H, CH_2_), 4.69 (t, *J* = 5.5 Hz, 2H, CH_2_), 3.77 (s, 2H, CH_2_). ^13^C NMR (151 MHz, DMSO-*d*6) δ/ppm 190.49, 158.83, 141.64, 130.95, 130.70, 125.46, 124.85, 124.83, 113.97, 111.64, 62.57, 47.69, 42.54, 35.75. Anal. calcd. for C_21_H_17_BrClN_5_O_2_ × H_2_O (*M*r = 504.76): C 49.97, H 3.79, N, 13.78; found: C 49.74, H 3.98, N 13.95.

#### 3.1.2. General Procedure for the Synthesis of Compounds **9a**–**10e** and **14a**–**15e**

A solution of an aldehyde **6**, **7**, **12 and 13** (1 mmol) appropriate benzene-1,2-diamine **9a**–**d** (1 mmol) and Na_2_S_2_O_5_ (0.5 mmol) in DMSO (15 mL) was heated at 165 °C for 15 min. The mixture was cooled down to room temperature. The addition of water (5 mL) resulted in precipitation. The resulting residues for compounds **10a**–**c**, **11a**–**c**, **12a**–**c**, **13a**–**c**, **14a**–**c**, **15a**–**c** were collected with filtration and products were obtained via recrystallization using methanol. The resulting residues for compounds **9d**–**e**, **10d**–**e**, **14d**–**e and 15d**–**e** were collected with filtration and products were obtained via recrystallization using methanol and converted to hydrochloride salts using anhydrous methanol saturated with HCl(g).

##### 4-(4-{[4-(1H-benzo[d]imidazol-2-yl)phenoxy]methyl}-1H-1,2,3-triazol-1-yl)-7-chloroquinoline (**9a**)

Compound **9a** was prepared using the above-described method from **6** (300 mg, 0.82 mmol), **8a** (90 mg, 0.82 mmol) and Na_2_S_2_O_5_ (76 mg, 0.41 mmol), as a light brown product (209 mg, 46%); mp = 143–145 °C. ^1^H NMR (600 MHz, DMSO-*d*6) δ/ppm 9.17 (d, *J* = 4.6 Hz, 1H, ArH), 9.05 (s, 1H, triaz.), 8.31 (d, *J* = 1.9 Hz, 1H, ArH), 8.19 (d, *J* = 8.8 Hz, 2H, ArH), 8.02 (d, *J* = 9.1 Hz, 1H, ArH), 7.90 (d, *J* = 4.6 Hz, 1H, ArH), 7.81 (dd, *J* = 2.0 Hz, *J* = 9.1 Hz, 1H, ArH), 7.71 (m, 2H, Ar), 7.46–7.33 (m, 4H, ArH), 5.48 (s, 2H, CH_2_). ^13^C NMR (151 MHz, DMSO-*d*6) δ/ppm 160.52, 152.37, 150.15, 149.36, 143.16, 140.31, 135.39, 129.03, 128.95, 127.14, 125.33, 123.67, 120.29, 119.67, 117.17, 115.60, 114.31, 61.14. Anal. calcd. for C_25_H_17_ClN_6_O (*M*r = 452.90): C 66.30, H 3.78, N 18.56; found: C 66.59, H 4.06, N 18.22.

##### 7-chloro-4-(4-{[4-(5-chloro-1H-benzo[d]imidazol-2-yl)phenoxy]methyl}-1H-1,2,3-triazol-1-yl)quinoline (**9b**)

Compound **9b** was prepared using the above-described method from **6** (330 mg, 0.9 mmol), **8b** (129 mg, 0.9 mmol) and Na_2_S_2_O_5_ (85 mg, 0.45 mmol), as a dark brown product (224 mg, 51%); mp = 168–170 °C. ^1^H NMR (600 MHz, DMSO-*d*6) δ/ppm 9.17 (d, *J* = 4.6 Hz, 1H, ArH), 9.03 (s, 1H, triaz.), 8.31 (s, 1H, ArH), 8.16 (d, *J* = 7.9 Hz, 2H, ArH), 8.01 (d, *J* = 9.1 Hz, 1H, ArH), 7.89 (d, *J* = 4.6 Hz, 1H, ArH), 7.81 (dd, *J* = 1.4 Hz, *J* = 9.1 Hz, 1H, ArH), 7.66 (s, 1H, Ar), 7.61 (d, *J* = 8.5 Hz, 1H, ArH), 7.33 (d, *J* = 7.9 Hz, 2H, ArH), 7.26 (d, *J* = 8.5 Hz, 1H, ArH), 5.45 (s, 2H, CH_2_). ^13^C NMR (151 MHz, DMSO-*d*6) δ/ppm 169.80, 152.35, 149.37, 143.29, 140.31, 135.37, 129.02, 128.39, 128.13, 127.07, 125.34, 122.38, 122.05, 120.31, 117.17, 115.87, 115.33, 61.05. Anal. calcd. for C_25_H_16_Cl_2_N_6_O × H_2_O (*M*r = 505.36): C 59.42, H 3.59, N 16.63; found: C 59.13, H 3.97, N 16.39.

##### 7-chloro-4-(4-{[4-(5-methoxy-1H--benzo[d]imidazol-2-yl)phenoxy]methyl}-1H-1,2,3-triazol-1-yl)quinoline (**9c**)

Compound **9c** was prepared using the above-described method from **6** (300 mg, 0.82 mmol), **8c** (113 mg, 0.82 mmol) and Na_2_S_2_O_5_ (76 mg, 0.41 mmol), as a light brown product (190 mg, 49%); mp = 85–86 °C. ^1^H NMR (600 MHz, DMSO-*d*6) δ/ppm 9.18 (d, *J* = 4.6 Hz, 1H, ArH), 9.04 (s, 1H, triaz.), 8.31 (d, *J* = 2.0 Hz, 1H, ArH), 8.15 (d, *J* = 7.2 Hz, 2H, ArH), 8.01 (d, *J* = 9.1 Hz, 1H, ArH), 7.90 (d, *J* = 4.6 Hz, 1H, ArH), 7.81 (dd, *J* = 2.0 Hz, *J* = 9.1 Hz, 1H, ArH), 7.57 (d, *J* = 8.7 Hz, 1H, ArH), 7.37 (d, *J* = 7.7 Hz, 2H, ArH), 5.46 (s, 2H, CH_2_), 3.84 (s, 3H, CH_2_). ^13^C NMR (151 MHz, DMSO-*d*6) δ/ppm 160.17, 156.59, 152.33, 149.40, 143.20, 140.28, 135.37, 129.03, 128.38, 128.16, 127.13, 125.34, 123.67, 120.37, 117.25, 115.55, 112.96, 112.94, 61.11, 55.64. Anal. calcd. for C_26_H_19_ClN_6_O_2_ × 0.5H_2_O (*M*r = 491.93): C 63.48, H 4.10, N 17.08; found: C 63.75, H 4.37, N 17.21.

##### 2-(4-{[1-(7-chloroquinolin-4-yl)-1H-1,2,3-triazol-4-yl]methoxy}phenyl)-1H-benzo[d]imidazol-5-carboximidamide trihydrochloride (**9d**)

Compound **9d** was prepared using the above-described method from **6** (300 mg, 0.82 mmol), **8d** (123 mg, 0.82 mmol) and Na_2_S_2_O_5_ (76 mg, 0.41 mmol), as a brown product (290 mg, 57%); mp = 240–242 °C. ^1^H NMR (600 MHz, DMSO-*d*6) δ/ppm 9.54 (s, 2H, NH), 9.28 (s, 2H, NH), 9.20 (s, 1H, ArH), 9.09 (s, 1H, triaz.), 8.52 (d, *J* = 7.2 Hz, 2H, ArH), 8.30 (s, 1H, ArH), 8.26 (s, 1H, ArH), 8.03 (d, *J* = 9.1 Hz, 1H, ArH), 7.94 (d, *J* = 8.5 Hz, 1H, ArH), 7.91 (d, *J* = 4.5 Hz, 1H, ArH), 7.87 (dd, *J* = 0.9 Hz, *J* = 8.5 Hz, 1H, ArH), 7.81 (dd, *J* = 1.4 Hz, *J* = 9.1 Hz, 1H, ArH), 7.45 (d, *J* = 8.8 Hz, 2H, ArH), 5.51 (s, 2H, CH_2_). ^13^C NMR (151 MHz, DMSO-*d*6) δ/ppm 165.51, 161.72, 152.20, 149.31, 142.96, 140.32, 135.42, 130.34, 129.08, 128.06, 127.26, 125.38, 124.45, 123.95, 120.39, 117.26, 115.79, 114.78, 114.21, 61.26, 55.64. Anal. calcd. for C_26_H_19_ClN_8_O × H_2_O × 3HCl (*M*r = 622.33): C 50.18, H 3.89, N 18.01; found: C 50.34, H 3.81, N 18.29.

##### 2-(4-{[1-(7-chloroquinolin-4-yl)-1H-1,2,3-triazol-4-yl]methoxy}phenyl)-N-propyl-1H-benzo[d]imidazol-5-carboximidamide trihydrochloride (**9e**)

Compound **9e** was prepared using the above-described method from **6** (300 mg, 0.82 mmol), **8e** (157 mg, 0.82 mmol) and Na_2_S_2_O_5_ (76 mg, 0.41 mmol), as a brown product (267 mg, 48%); mp = 228–230 °C. ^1^H NMR (600 MHz, DMSO-*d*6) δ/ppm 9.99 (t, *J* = 4.1 Hz, 1H, NH), 9.64 (s, 1H, NH), 9.18 (d, *J* = 4.6 Hz, 1H, ArH), 9.17 (s, 1H, NH), 9.09 (s, 1H, triaz.), 8.54 (d, *J* = 8.7 Hz, 2H, ArH), 8.32 (d, *J* = 2.1 Hz, 1H, ArH), 8.18 (d, *J* = 1.1 Hz, 1H, ArH), 8.03 (d, *J* = 9.1 Hz, 1H, ArH), 7.95 (d, *J* = 8.5 Hz, 1H, ArH), 7.91 (d, *J* = 4.6 Hz, 1H, ArH), 7.83 (dd, *J* = 2.1 Hz, *J* = 9.1 Hz, 1H, ArH), 7.79 (dd, *J* = 1.1 Hz, *J* = 8.5 Hz, 1H, ArH), 7.47 (d, *J* = 9.0 Hz, 2H, ArH), 5.52 (s, 2H, CH_2_), 3.43 (m, 2H, CH_2_), 1.71 (m, 2H, CH_2_), 0.99 (t, *J* = 7.2 Hz, 1H, CH_2_). ^13^C NMR (151 MHz, DMSO-*d*6) δ/ppm 162.74, 161.75, 152.31, 151.69, 149.26, 142.96, 140.37, 135.44, 130.34, 129.10, 128.06, 127.27, 125.37, 125.07, 124.62, 123.95, 120.31, 117.20, 115.82, 114.67, 114.18, 114.21, 61.25, 44.38. 20.76, 11.19. Anal. calcd. for C_29_H_25_ClN_8_O × 1.5H_2_O × 3HCl (*M*r = 673.42): C 51.72, H 4.64, N 16.64; found: C 51.40, H 4.89, N 16.26.

##### 4-(4-{[4-(1H-benzo[d]imidazol-2-yl)-2-bromophenoxy]methyl}-1H-1,2,3-triazol-1-yl)-7-chloroquinoline (**10a**)

Compound **10a** was prepared using the above-described method from **7** (300 mg, 0.68 mmol), **8a** (74 mg, 0.68 mmol) and Na_2_S_2_O_5_ (65 mg, 0.34 mmol), as a light brown product (167 mg, 47%); mp = 252–254 °C. ^1^H NMR (600 MHz, DMSO-*d*6) δ/ppm 12.91 (s, 1H, NH), 9.18 (d, *J* = 4.5 Hz, 1H, ArH), 9.04 (s, 1H, triaz.), 8.44 (s, 1H, ArH), 8.31 (d, *J* = 2.1 Hz, 1H, ArH), 8.22 (d, *J* = 8.2 Hz, 1H, ArH), 8.01 (d, *J* = 9.1 Hz, 1H, ArH), 7.91 (d, *J* = 4.6 Hz, 1H, ArH), 7.81 (dd, *J* = 2.1 Hz, *J* = 9.1 Hz, 1H, ArH), 7.65 (d, *J* = 8.5 Hz, 1H, ArH), 7.63 (d, *J* = 8.5 Hz, 1H, ArH), 7.53 (s, 1H, Ar), 7.21 (s, 2H, Ar), 5.55 (s, 2H, CH_2_). ^13^C NMR (151 MHz, DMSO-*d*6) δ/ppm 155.39, 152.35, 149.37, 143.72, 142.96, 140.27, 135.37, 130.91, 129.03, 128.15, 127.19, 127.17, 125.32, 124.64, 122.49, 121.69, 120.32, 118.71, 117.22, 114.66, 111.60, 111.24, 62.15. Anal. calcd. for C_25_H_16_ClBrN_6_O × H_2_O (*M*r = 549.81): C 54.61, H 3.30, N 15.29; found: C 54.98, H 3.09, N 15.20.

##### 4-(4-{[2-bromo-4-(5-chloro-1H-benzo[d]imidazol-2-yl)phenoxy]methyl}-1H-1,2,3-triazol-1-yl)-7-chloroquinoline (**10b**)

Compound **10b** was prepared using the above-described method from **7** (300 mg, 0.68 mmol), **8b** (96 mg, 0.68 mmol) and Na_2_S_2_O_5_ (65 mg, 0.34 mmol), as a brown product (218 mg, 54%); mp = 250–252 °C. ^1^H NMR (600 MHz, DMSO-*d*6) δ/ppm 9.18 (d, *J* = 4.5 Hz, 1H, ArH), 9.04 (s, 1H, triaz.), 8.42 (d, *J* = 1.5 Hz, 1H, ArH), 8.30 (d, *J* = 2.1 Hz, 1H, ArH), 8.21 (dd, *J* = 1.4 Hz, *J* = 8.6 Hz, 1H), 8.01 (d, *J* = 9.1 Hz, 1H, ArH), 7.91 (d, *J* = 4.6 Hz, 1H, ArH), 7.81 (dd, *J* = 2.1 Hz, *J* = 9.1 Hz, 1H), 7.64 (d, *J* = 8.7 Hz, 2H, ArH), 7.60 (d, *J* = 8.5 Hz, 1H, ArH), 7.23 (dd, *J* = 2.1 Hz, *J* = 8.5 Hz, 1H), 5.55 (s, 2H, CH_2_). ^13^C NMR (151 MHz, DMSO-*d*6) δ/ppm 155.71, 152.35, 149.36, 142.91, 140.26, 135.36, 131.10, 129.02, 128.15, 127.43, 127.20, 126.47, 125.31, 123.93, 122.41, 120.29, 117.19, 114.65, 111.63, 111.24, 62.16. Anal. calcd. for C_25_H_16_ClBrN_6_O × 1.5H_2_O (*M*r = 593.26): C 50.61, H 3.06, N 14.17; found: C 50.89, H 3.11, N 14.33.

##### 4-(4-{[2-bromo-4-(5-methoxy-1H-benzo[d]imidazol-2-yl)phenoxy]methyl}-1H-1,2,3-triazol-1-yl)-7-chloroquinoline (**10c**)

Compound **10c** was prepared using the above-described method from **7** (300 mg, 0.68 mmol), **8c** (92 mg, 0.68 mmol) and Na_2_S_2_O_5_ (65 mg, 0.34 mmol), as a light brown product (245 mg, 64%); mp = 170–172 °C. ^1^H NMR (600 MHz, DMSO-*d*6) δ/ppm 9.18 (d, *J* = 4.6 Hz, 1H, ArH), 9.05 (s, 1H, triaz.), 8.43 (s, 1H, Ar), 8.31 (d, *J* = 2.0 Hz, 1H, ArH), 8.19 (d, *J* = 8.4 Hz, 1H, ArH), 8.01 (d, *J* = 9.1 Hz, 1H, ArH), 7.91 (d, *J* = 4.6 Hz, 1H, ArH), 7.81 (dd, *J* = 2.0 Hz, *J* = 9.1 Hz, 1H, ArH), 7.69 (d, *J* = 8.4 Hz, 1H, ArH), 7.58 (d, *J* = 8.7 Hz, 1H, ArH), 7.15 (s, 1H, Ar), 6.97 (dd, *J* = 1.7 Hz, *J* = 8.4 Hz, 1H, ArH), 5.57 (s, 2H, CH_2_), 3.85 (s, 3H, CH_2_). ^13^C NMR (151 MHz, DMSO-*d*6) δ/ppm 156.69, 156.11, 152.36, 149.37, 142.82, 140.25, 135.38, 131.22, 129.04, 128.16, 127.59, 127.24, 125.30, 120.29, 117.21, 114.74, 113.14, 111.75, 62.22, 55.64. Anal. calcd. for C_26_H_18_BrClN_6_O_2_ × H_2_O (*M*r = 579,83): C 53.86, H 3.48, N 14.49; found: C 54.02, H 3.69, N 14.41.

##### 2-(3-bromo-4-{[1-(7-chloroquinolin-4-yl)-1H-1,2,3-triazol-4-yl]methoxy}phenyl)-1H-benzo[d]imidazol-5-carboximidamide trihydrochloride (**10d**)

Compound **10d** was prepared using the above-described method from **7** (324 mg, 0.73 mmol), **8d** (109 mg, 0.73 mmol) and Na_2_S_2_O_5_ (69 mg, 0.36 mmol), as a dark brown product (263 mg, 63%); mp = 200–202 °C. ^1^H NMR (600 MHz, DMSO-*d*6) δ/ppm 9.41 (s, 2H, NH), 9.17 (d, *J* = 4.6 Hz, 1H, ArH), 9.09 (s, 2H, NH), 9.06 (s, 1H, triaz.), 8.59 (d, *J* = 2.1 Hz, 1H, ArH), 8.40 (dd, *J* = 2.0 Hz, *J* = 8.6 Hz, 1H, ArH), 8.30 (d, *J* = 2.1 Hz, 1H, ArH), 8.18 (d, *J* = 1.2 Hz, 1H, ArH), 8.01 (d, *J* = 9.1 Hz, 1H, ArH), 7.91 (d, *J* = 4.6 Hz, 1H, ArH), 7.84 (d, *J* = 8.5 Hz, 1H, ArH), 7.81 (dd, *J* = 2.1 Hz, *J* = 9.1 Hz, 1H, ArH), 7.74 (dd, *J* = 1.6 Hz, *J* = 8.5 Hz, 1H, ArH), 7.70 (d, *J* = 8.8 Hz, 1H, ArH), 5.58 (s, 2H, CH_2_). ^13^C NMR (151 MHz, DMSO-*d*6) δ/ppm 165.55, 157.53, 152.14, 149.19, 142.65, 140.35, 135.47, 132.67, 129.38, 129.12, 127.97, 127.36, 125.38, 124.30, 123.80, 120.37, 119.05, 117.28, 115.10, 114.73, 114.42, 111.91, 62.32. Anal. calcd. for C_26_H_18_BrClN_8_O × 2H_2_O × 3HCl (*M*r = 719.24): C 43.42, H 3.50, N 15.58; found: C 43.11, H 3.87, N 15.26.

##### 2-(3-bromo-4-{[1-(7-chloroquinolin-4-yl)-1H-1,2,3-triazol-4-yl]methoxy}phenyl)-N-propyl-1H-benzo[d]imidazol-5-carboximidamide trihydrochloride (**10e**)

Compound **10e** was prepared using the above-described method from **7** (300 mg, 0.67 mmol), **8e** (128 mg, 0.67 mmol) and Na_2_S_2_O_5_ (64 mg, 0.67 mmol), as a dark brown product (214 mg, 52%); mp = 213–214 °C. ^1^H NMR (600 MHz, DMSO-*d*6) δ/ppm 9.86 (t, *J* = 4.5 Hz, 1H, NH), 9.53 (s, 1H, NH), 9.18 (d, *J* = 4.6 Hz, 1H, ArH), 9.07 (brs, 2H, NH + triaz.), 8.67 (d, *J* = 1.3 Hz, 1H, ArH), 8.48 (d, *J* = 8.5 Hz, 1H, ArH), 8.32 (d, *J* = 2.1 Hz, 1H, ArH), 8.12 (s, 1H, ArH), 8.02 (d, *J* = 9.1 Hz, 1H, ArH), 7.92 (d, *J* = 4.6 Hz, 1H, ArH), 7.88 (d, *J* = 8.5 Hz, 1H, ArH), 7.83 (dd, *J* = 2.1 Hz, *J* = 9.1 Hz, 1H, ArH), 7.74 (d, *J* = 9.1 Hz, 1H, ArH), 7.70 (dd, *J* = 1.1 Hz, *J* = 8.5 Hz, 1H, ArH), 5.60 (s, 2H, CH_2_), 3.42 (m, 2H, CH_2_), 1.71 (m, 2H, CH_2_), 0.99 (t, *J* = 7.1 Hz, 1H, CH_2_). ^13^C NMR (151 MHz, DMSO-*d*6) δ/ppm 162.70, 157.64, 152.23, 150.56, 149.13, 142.63, 140.39, 135.48, 132.77, 129.50, 129.14, 127.95, 127.38, 125.37, 125.10, 124.61, 120.30, 117.23, 114.91, 114.75, 114.31, 111.94, 62.33, 44.38. 26.15, 20.78, 11.19. Anal. Calcd. For C_29_H_25_ClN_8_O × 0.5H_2_O × 3HCl (*M*r = 734.30): C 47.43, H 3.84, N 15.26; found: C 47.03, H 3.99, N 14.91.

##### N-[2-(4-{[4-(1H-benzo[d]midazole-2-yl)phenoxy]methyl}-1H-1,2,3-triazol-1-yl)ethyl]-7-chloroquinolin-4-amine (**14a**)

Compound **14a** was prepared using the above-described method from **12** (300 mg, 0.74 mmol), **8a** (80 mg, 0.74 mmol) and Na_2_S_2_O_5_ (70 mg, 0.37 mmol), as a light brown product (160 mg, 44%); mp = 221–223 °C. ^1^H NMR (600 MHz, DMSO-*d*6) δ/ppm 8.55 (brs, 2H, ArH + triaz.), 8.31 (brs, 2H, ArH), 8.13 (brs, 2H, ArH), 7.63 (brs, 2H, ArH), 7.61 (brs, 2H, ArH + NH), 7.18 (brs, 2H, ArH), 7.16 (brs, 2H, ArH), 6.91 (brs, 1H, ArH), 5.21 (s, 2H, CH_2_), 4.72 (s, 2H, CH_2_), 3.94 (s, 2H, CH_2_). ^13^C NMR (151 MHz, DMSO-*d*6) δ/ppm 157.29, 142.52, 135.72, 127.83, 126.09, 125.28, 123.08, 121.83, 115.03, 114.84, 61.12, 47.67, 42.77. Anal. calcd. for C_27_H_22_ClN_7_O × 0.5H_2_O (*M*r = 504.97): C 64.22, H 4.59, N 19.42; found: C 63.89, H 4.21, N 19.16.

##### 7-chloro-N-[2-(4-{[4-(5-chloro-1H-benzo[d]imidazol-2-yl)phenoxy]methyl}-1H-1,2,3-triazol-1-yl)ethyl]quinolin-4-amine (**14b**)

Compound **14b** was prepared using the above-described method from **12** (300 mg, 0.74 mmol), **8b** (105 mg, 0.74 mmol) and Na_2_S_2_O_5_ (70 mg, 0.37 mmol), as a brown product (160 mg, 44%); mp = 213–215 °C. ^1^H NMR (600 MHz, DMSO-*d*6) δ/ppm 8.43 (s, 1H, ArH), 8.34 (brs, 1H, ArH), 8.31 (s, 1H, ArH), 8.10 (brs, 2H, ArH), 7.89 (brs, 1H, ArH), 7.62 (d, *J* = 8.8 Hz, 2H, ArH), 7.58 (brs, 1H, ArH), 7.19 (d, *J* = 8.8 Hz, 2H, ArH), 7.58 (brs, 1H, ArH), 6.77 (brs, 1H, ArH), 5.21 (s, 2H, CH_2_), 4.72 (t, *J* = 5.7 Hz, 2H, CH_2_), 3.94 (d, *J* = 4.5 Hz, 2H, CH_2_). ^13^C NMR (151 MHz, DMSO-*d*6) δ/ppm 159.54, 152.67, 147.32, 142.42, 135.85, 128.12, 126.04, 125.80, 125.26, 124.65, 123.39, 122.45, 121.96, 115.08, 115.87, 115.33, 61.16, 55.99, 42.68. Anal. calcd. for C_27_H_21_Cl_2_N_7_O × H_2_O (*M*r = 548.42): C 59.13, H 4.23, N 17.88; found: C 59.21, H 4.54, N 17.55.

##### 7-chloro-N-[2-(4-{[4-(5-methoxy-1H-benzo[d]imidazol-2-yl)phenoxy]methyl}-1H-1,2,3-triazol-1-yl)ethyl]quinolin-4-amine (**14c**)

Compound **14c** was prepared using the above-described method from **12** (300 mg, 0.74 mmol), **8c** (102 mg, 0.74 mmol) and Na_2_S_2_O_5_ (70 mg, 0.37 mmol), as a brown product (206 mg, 53%); mp = 150–152 °C. ^1^H NMR (600 MHz, DMSO-*d*6) δ/ppm 9.21 (brs, 1H, ArH), 8.52 (brs, 1H, ArH), 8.39 (brs, 1H, ArH), 8.32 (s, 1H, ArH), 8.06 (brs, 2H, ArH), 7.92 (brs, 1H, ArH), 7.75 (d, *J* = 8.8 Hz, 1H, ArH), 7.48 (brs, 1H, ArH), 7.15 (d, *J* = 6.7 Hz, 2H, ArH), 7.09 (brs, 1H, ArH), 6.82 (d, *J* = 8.0 Hz, 2H, ArH), 5.20 (s, 2H, CH_2_), 4.75 (t, *J* = 5.5 Hz, 2H, CH_2_), 4.05 (s, 2H, CH_2_), 3.80 (s, 3H, CH_3_). ^13^C NMR (151 MHz, DMSO-*d*6) δ/ppm 159.17, 155.72, 155.06, 143.90, 142.54, 139.20, 137.72, 127.74, 126.93, 125.33, 125.20, 122.70, 119.97, 115.61, 115.02, 111.38, 61.11, 55.44, 47.77, 42.92. Anal. calcd. for C_28_H_24_ClN_7_O_2_ × 1.5H_2_O (*M*r = 553.01): C 60.81, H 4.92, N 17.73; found: C 60.43, H 4.99, N 17.40.

##### 2-{4-[(1-{2-[(7-chloroquinolin-4-yl)amino]ethyl}-1H-1,2,3-triazol-4-yl)methoxy]phenyl}-1H-benzo[d]imidazol-5-carboximidamide trihydrochloride (**14d**)

Compound **14d** was prepared using the above-described method from **12** (300 mg, 0.74 mmol), **8d** (111 mg, 0.74 mmol) and Na_2_S_2_O_5_ (70 mg, 0.37 mmol), as a dark brown product (267 mg, 49%); mp = 227–229 °C. ^1^H NMR (600 MHz, DMSO-*d*6) δ/ppm 14.45 (s, 1H, NH), 9.71 (s, 1H, NH), 9.56 (s, 2H, NH), 9.24 (s, 2H, NH), 8.60 (d, *J* = 7.2 Hz, 1H, ArH), 8.47 (brs, 1H, ArH), 8.43 (brs, 1H, ArH), 8.41 (brs, 2H, ArH), 8.05 (brs, 1H, ArH), 7.94 (brs, 1H, ArH), 7.87 (brs, 1H, ArH), 7.74 (d, *J* = 7.2, 1H, ArH), 7.30 (d, *J* = 5.6 Hz, 1H, ArH), 6.76 (brs, 2H, ArH), 5.26 (s, 2H, CH_2_), 4.78 (s, 2H, CH_2_), 4.07 (s, 2H, CH_2_). ^13^C NMR (151 MHz, DMSO-*d*6) δ/ppm 165.41, 161.91, 159.17, 155.62, 142.85, 142.03, 138.36, 137.96, 130.40, 126.98, 125.75, 124.79, 124.34, 118.95, 115.72, 115.36, 115.11, 114.63, 114.23, 98.43, 61.44, 47.91, 42.99. Anal. calcd. for C_28_H_24_ClN_9_O × 2H_2_O × 3HCl (*M*r = 683.42): C 49.21, H 4.57, N 18.45; found: C 49.56, H 4.70, N 18.17.

##### 2-{4-[(1-{2-[(7-chloroquinolin-4-yl)amino]ethyl}-1H-1,2,3-triazol-4-yl)methoxy]phenyl}-N-propyl-1H-benzo[d]imidazol-5-carboximidamide trihydrochloride (**14e**)

Compound **14e** was prepared using the above-described method from **12** (300 mg, 0.74 mmol), **8e** (142 mg, 0.74 mmol) and Na_2_S_2_O_5_ (70 mg, 0.37 mmol), as a dark brown product (210 mg, 49%); mp = 209–211 °C. ^1^H NMR (600 MHz, DMSO-*d*6) δ/ppm 14.31 (s, 1H, NH), 9.88 (s, 1H, NH), 9.62 (t, *J* = 5.1 Hz, 1H, NH), 9.55 (s, 1H, NH), 9.08 (s, 1H, NH), 8.54 (m, 2H, ArH), 8.38 (brs, 2H, ArH), 8.34 (brs, 1H, ArH), 8.13 (brs, 1H, ArH), 8.03 (d, *J* = 1.7, 1H, ArH), 7.91 (d, *J* = 8.5, 1H, ArH), 7.78 (dd, *J* = 1.5 Hz, *J* = 9.1 Hz, 1H, ArH), 7.72 (d, *J* = 8.2, 1H, ArH), 7.29 (d, *J* = 8.2 Hz, 2H, ArH), 6.80 (d, *J* = 7.0 Hz, 2H, ArH), 5.26 (s, 2H, CH_2_), 4.79 (t, *J* = 5.1 Hz, 2H, CH_2_), 4.08 (m, 2H, CH_2_), 3.42 (m, 2H, CH_2_), 1.71 (m, 2H, CH_2_), 0.94 (t, *J* = 7.3 Hz, 2H, CH_2_). ^13^C NMR (151 MHz, DMSO-*d*6) δ/ppm 162.75, 161.74, 155.64, 142.90, 142.10, 138.36, 138.00, 130.20, 127.01, 125.67, 125.15, 124.66, 119.0, 115.69, 115.36, 114.69, 114.21, 98.45, 61.38, 47.86, 44.38, 42.97, 20.75, 11.21. Anal. calcd. for C_31_H_30_ClN_9_O × 1.5H_2_O × 2HCl (*M*r = 716.49): C 51.97, H 5.06, N 17.59; found: C 51.76, H 5.42, N 17.23.

##### N-[2-(4-{[4-(1H-benzo[d]imidazol-2-yl)-2-bromophenoxy]methyl}-1H-1,2,3-triazol-1-yl)ethyl]-7-chloroquinolin-4-amine (**15a**)

Compound **15a** was prepared using the above-described method from **13** (250 mg, 0.51 mmol), **8a** (55 mg, 0.51 mmol) and Na_2_S_2_O_5_ (50 mg, 0.26 mmol), as a brown product (156 mg, 53%); mp = 155–157 °C. ^1^H NMR (600 MHz, DMSO-*d*6) δ/ppm 8.47 (brs, 1H, ArH), 8.38 (brs, 1H, ArH), 8.31 (s, 1H, ArH), 8.19 (d, *J* = 8.6 Hz, 1H, ArH), 8.14 (dd, *J* = 0.9 Hz, *J* = 8.6 Hz, 1H, ArH), 7.82 (brs, 1H, ArH), 7.63 (brs, 1H, ArH), 7.58 (brs, 2H, ArH), 7.49 (d, *J* = 8.6 Hz, 2H, ArH), 7.19 (m, 2H, ArH), 6.60 (brs, 2H, ArH), 5.32 (s, 2H, CH_2_), 4.71 (t, *J* = 5.9 Hz, 2H, CH_2_), 3.83 (m, 2H, CH_2_), 3.80 (s, 3H, CH_3_). ^13^C NMR (151 MHz, DMSO-*d*6) δ/ppm 155.39, 150.82, 150.00, 141.99, 133.84, 130.81, 127.09, 125.31, 124.64, 124.34, 124.18, 122.06, 115.04, 114.30, 111.41, 62.33, 47.87, 42.42, 40.38. Anal. calcd. for C_27_H_21_BrClN_7_O × 1.5H_2_O (*M*r = 601.88): C 53.88, H 4.02, N 16.29; found: C 54.11, H 4.23, N 16.44.

##### N-[2-(4-{[2-bromo-4-(5-chloro-1H-benzo[d]imidazol-2-yl)phenoxy]methyl}-1H-1,2,3-triazol-1-yl)ethyl]-7-chloroquinolin-4-amine (**15b**)

Compound **15b** was prepared using the above-described method from **13** (300 mg, 0.62 mmol), **8b** (88 mg, 0.62 mmol) and Na_2_S_2_O_5_ (59 mg, 0.31 mmol), as a brown product (195 mg, 50%); mp = 100–102 °C. ^1^H NMR (600 MHz, DMSO-*d*6) δ/ppm 8.36 (brs, 2H, ArH), 8.31 (s, 3H, ArH), 8.12 (d, *J* = 7.9 Hz, 1H, ArH), 7.88 (brs, 1H, ArH), 7.62 (brs, 1H, ArH), 7.60 (d, *J* = 8.8 Hz, 2H, ArH), 7.58 (brs, 1H, ArH), 7.48 (d, *J* = 8.5 Hz, 1H, ArH), 7.22 (dd, *J* = 0.9 Hz, 8.4 Hz, 1H, ArH), 6.74 (brs, 1H, ArH), 5.32 (s, 2H, CH_2_), 4.73 (t, *J* = 5.0 Hz, 2H, CH_2_), 3.33 (t, *J* = 4.0 Hz, 2H, CH_2_). ^13^C NMR (151 MHz, DMSO-*d*6) δ/ppm 155.60, 150.84, 141.98, 135.34, 130.90, 127.23, 126.33, 125.82, 125.41, 125.09, 123.85, 122.29, 114.27, 111.40, 62.30, 47.79, 42.70. Anal. calcd. for C_27_H_20_BrCl_2_N_7_O × H_2_O (*M*r = 627.32): C 51.69, H 3.53, N 15.63; found: C 51.76, H 3.90, N 15.27.

##### N-[2-(4-{[2-bromo-4-(5-methoxy-1H-benzo[d]imidazol-2-yl)phenoxy]methyl}-1H-1,2,3-triazol-1-yl)ethyl]-7-chloroquinolin-4-amine (**15c**)

Compound **15c** was prepared using the above-described method from **13** (266 mg, 0.55 mmol), **8c** (75 mg, 0.55 mmol) and Na_2_S_2_O_5_ (52 mg, 0.31 mmol), as a brown product (186 mg, 55%); mp = 92–94 °C. ^1^H NMR (600 MHz, DMSO-*d*6) δ/ppm 9.24 (s, 1H, ArH), 8.56 (brs, 1H, ArH), 8.41 (d, *J* = 6.0 Hz, 1H, ArH), 8.33 (brs, 1H, ArH), 8,32 (s, 1H, ArH), 8.07 (d, *J* = 7.4 Hz, 1H, ArH), 7.92 (brs, 1H, ArH), 7.77 (d, *J* = 9.0 Hz, 1H, ArH), 7.48 (d, *J* = 8.2 Hz, 2H, ArH), 7.45 (d, *J* = 8.5 Hz, 1H, ArH), 7.07 (s, 1H, ArH), 6.86 (brs, 1H, ArH), 6.83 (dd, *J* = 1.6 Hz, 8.5, 1H, ArH), 5.30 (s, 2H, CH_2_), 4.77 (t, *J* = 5.7 Hz, 2H, CH_2_), 4.06 (q, *J* = 5.5 Hz, 2H, CH_2_), 3.80 (s, 3H, CH_3_). ^13^C NMR (151 MHz, DMSO-*d*6) δ/ppm 155.81, 155.10, 143.63, 142.16, 137.81, 130.49, 127.01, 126.70, 125.47, 125.31, 124.48, 124.46, 114.27, 111.60, 111.34, 62.25, 55.44, 47.79, 42.93. Anal. calcd. for C_28_H_23_BrClN_7_O_2_ × H_2_O (*M*r = 622.90): C 53.99, H 4.05, N 15.74; found: C 54.26, H 4.41, N 15.41.

##### 2-{3-bromo-4-[(1-{2-[(7-chloroquinolin-4-yl)amino]ethyl}-1H-1,2,3-triazol-4-yl)methoxy]phenyl}-1H-benzo[d]imidazol-5-carboximidamide trihydrochloride (**15d**)

Compound **15d** was prepared using the above-described method from **13** (345 mg, 0.71 mmol), **8d** (106 mg, 0.71 mmol) and Na_2_S_2_O_5_ (67 mg, 0.37 mmol), as a dark brown product (260 mg, 50%); mp = 215–217 °C. ^1^H NMR (600 MHz, DMSO-*d*6) δ/ppm 14.45 (s, 1H, NH), 9.78 (t, *J* = 5.5 Hz, 1H, NH), 9.54 (s, 2H, NH), 9.24 (s, 2H, NH), 8.67 (brs, 2H, ArH), 8.64 (d, *J* = 9.1 Hz, 1H, ArH), 8.52–8.43 (m, 2H, ArH), 8.41 (brs, 1H, ArH), 8.24 (brs, 1H, ArH), 8.08 (d, *J* = 2.0 Hz, 1H, ArH), 7.91 (d, *J* = 8.5 Hz, 1H, ArH), 7.83 (d, *J* = 8.3 Hz, 1H, ArH), 7.75 (dd, *J* = 1.9 Hz, 9.1 Hz, 1H, ArH), 7.57 (d, *J* = 8.5 Hz, 1H, ArH), 6.77 (d, *J* = 7.1 Hz, 1H, ArH), 5.36 (s, 2H, CH_2_), 4.82 (t, *J* = 5.5 Hz, 2H, CH_2_), 4.36 (s, 2H, CH_2_). ^13^C NMR (151 MHz, DMSO-*d*6) δ/ppm 165.53, 157.41, 155.61, 142.76, 141.79, 138.39, 137.97, 132.53, 129.17, 126.95, 125.80, 124.17, 123.65, 118.94, 115.36, 115.06, 114.42, 111.72, 98.38, 62.49, 47.91, 42.96. Anal. calcd. for C_28_H_23_BrClN_9_O × 0.5H_2_O × 3HCl (*M*r = 735.29): C 45.74, H 3.70, N 17.14; found: C 45.41, H 3.94, N 16.92.

##### 2-{3-bromo-4-[(1-{2-[(7-chloroquinolin-4-yl)amino]ethyl}-1H-1,2,3-triazol-4-yl)methoxy]phenyl}-N-propyl-1H-benzo[d]imidazol-5-carboximidamide trihydrochloride (**15e**)

Compound **15e** was prepared using the above-described method from **13** (300 mg, 0.62 mmol), **8e** (119 mg, 0.62 mmol) and Na_2_S_2_O_5_ (59 mg, 0.31 mmol), as a dark brown product (273 mg, 56%); mp = 169–171 °C. ^1^H NMR (600 MHz, DMSO-*d*6) δ/ppm 14.44 (s, 1H, NH), 9.87 (s, 1H, NH), 9.71 (t, *J* = 5.2 Hz, 1H, NH), 9.54 (s, 1H, NH), 9.08 (s, 1H, NH), 8.62 (brs, 1H, ArH), 8.60 (d, *J* = 9.1, 1H, ArH), 8.50 (d, *J* = 6.6, 1H, ArH), 8.40 (brs, 2H, ArH), 8.13 (brs, 1H, ArH), 8.06 (d, *J* = 1.8, 1H, ArH), 7.88 (d, *J* = 7.7, 1H, ArH), 7.76 (dd, *J* = 1.8 Hz, *J* = 9.1 Hz, 1H, ArH), 7.70 (d, *J* = 8.2, 1H, ArH), 7.56 (d, *J* = 8.2 Hz, 1H, ArH), 6.79 (d, *J* = 7.0 Hz, 1H, ArH), 5.36 (s, 2H, CH_2_), 4.81 (t, *J* = 5.2 Hz, 2H, CH_2_), 4.08 (d, *J* = 5.2 Hz, 2H, CH_2_), 3.42 (q, *J* = 6.5 Hz, 2H, CH_2_), 1.75–1.66 (m, 2H, CH_2_), 0.99 (t, *J* = 7.3 Hz, 2H, CH_2_). ^13^C NMR (151 MHz, DMSO-*d*6) δ/ppm 163.19, 155.67, 143.04, 138.35, 138.10, 128.56, 128.28, 127.09, 127.71, 125.49, 119.10, 114.41, 111.59, 98.53, 62.41, 47.85, 44.29, 42.96, 20.78, 11.20. Anal. calcd. for C_31_H_29_BrClN_9_O × 0.75H_2_O × 3HCl (*M*r = 781.87): C 47.62, H 4.32, N 16.12; found: C 47.33, H 3.94, N 15.97

### 3.2. Biological Activity

#### 3.2.1. Cell Lines and Cell Culturing

The effect of the new synthesized compounds was tested on five human tumor cell lines, HeLa (human cervical adenocarcinoma; from ATCC), CaCo-2 (human colorectal adenocarcinoma), HL-60 (acute promyelocytic leukemia), HuT78 (T-cell lymphoma) and THP-1 (acute monocytic leukemia), and on one non-tumor cell line, MRC-5 (human fetal lung fibroblasts). The MRC-5 cells were used between 24 and 26 passages. The cells were cultured in two different types of media: DMEM (Gibco, EU) and RPMI 1640 (Gibco, EU). Both media were supplemented with 2 mM glutamine, fetal bovine serum (10%; heat inactivated) and antibiotics (100 U penicillin and 0.1 mg streptomycin). RPMI 1640 was additionally supplemented with 10 mM HEPES and 1 mM sodium pyruvate. The cells growing in a monolayer were cultured in DMEM, while the cells growing in suspension were cultured in RPMI 1640. The cells were grown in humidified atmosphere under the conditions of 37 °C/5% CO_2_ gas in the CO_2_ incubator (IGO 150 CELLlife^TM^, JOUAN, Thermo Fisher Scientific, Waltham, MA, USA).

#### 3.2.2. Proliferation Assay

Growth-inhibitory activity was assessed using a slightly modified procedure based on the National Cancer Institute’s protocol [55]. Briefly, the cells were seeded in 96-well microtiter plates and incubated for 24 h. They were then treated with 10^−7^ to 10^−4^ M concentrations of the tested compounds for an additional 72 h. After the treatment period, the effects of the tested compounds on the growth rate of the cells were examined using the MTT assay [56]. Absorbance was measured at 595 nm using a microplate reader. The IC_50_ value, which represents a 50% inhibition of cell growth, and QC calculation were performed using the GraphPadPrism and Excel software. The selectivity index was calculated according to the following formula:

$$SI=\frac{{IC}_{50}\;value\;of\;normal\;cell\;line}{{IC}_{50}\;value\;of\;cancer\;cell\;line}$$

The effect of each concentration was analyzed by plotting the logarithm of the concentration of the evaluated compound against the corresponding percentage inhibition value using least squares.

#### 3.2.3. Cell Cycle Analysis

HuT78 cells (1 × 10^5^ cells/mL) were seeded in 24-well plates (Falcon, Durham, SAD) and treated with previously determined IC_50_ values for compounds **10e**, **14e**, and **15e** (5 × 10^−6^ mol/dm^3^) and **9c** (0.5 × 10^−6^ mol/dm^3^). The cells were exposed to the compounds for 24 h, collected, washed with PBS, fixed with 70% ethanol and stored at −20 °C until analysis. On the day of analysis, the cell pellets were washed twice with PBS, resuspended in 1 mg/mL PI and 0.2 mg/mL RNase A and left in a dark/cold atmosphere for 30 min. The stained cells were analyzed using FACSCalibur (Becton Dickinson, Franklin Lakes, NJ, USA) flow cytometer. Tests were performed in duplicate and repeated twice. FlowJo software, version 10.2., was adapted for the analysis of DNA histograms.

STATISTICA 13.5 (TIBCO Software Inc., Tulsa, USA) was used for the statistical analysis of the results. Student’s t test was used for data analysis. Differences were considered statistically significant at *p* < 0.05.

### 3.3. Computational Methods

#### 3.3.1. Calculation of ADME Properties

SwissADME web tool (http://www.swissadme.ch, accessed on 25 August 2023) gives free access to a pool of fast yet robust predictive models for physicochemical properties, pharmacokinetics, drug-likeness and medicinal chemistry friendliness parameters through the input of molecular structure [49].

#### 3.3.2. Molecular Docking

Molecular docking of quinoline–benzimidazole hybrids, which exhibited antiproliferative activities, was performed on the MAP3K TAO2 kinase. The crystal structure of the MAP3K TAO2 kinase domain was taken with bounded inhibitor staurosporine from the PDB base (PDB ID: 2GCD). Protein structure was prepared sing BIOVIA Discovery Studio Visualizer 4.5 (Dassault Systèmes, Paris, France). The 3D structures of ligands were optimized using Spartan ’08 (Wavefunction, Inc.; Irvine, CA, USA, 2009), using the molecular mechanics force field (MM+) [57], and subsequently via the semiempirical AM1 method [58]. The molecular-docking-optimized molecular structures of 12 compounds were performed with iGEMDOCK (BioXGEM, Hsinchu, Taiwan) using generic evolutionary method (GA). The GA parameters were set as follows: population size 200; generations 70; number of poses 3; and binding site radius 8 Å). After each compound was docked into the binding site, iGEMDOCK generated protein–compound interaction profiles of electrostatic (E), hydrogen-bonding (H), and van der Waals (V) interactions. iGEMDOCK infers pharmacological interactions and clusters for post-screening analysis based on these profiles and compound structures. Finally, the docked compounds were ranked by total energy of a predicted pose in the binding site that is defined as: (*E*/kcal mol^−1^) is: *E* = vdW + Hbond + Elec, where the vdW is van der Waal energy, Hbond is the hydrogen bonding energy, and Elect terms are electro statistic energy [59].

## 4. Conclusions

The targeted 1,2,3-triazole-containing quinoline–benzimidazole hybrids **9a**–**10e** and **14b**–**15d** were synthesized via the coupling of phenylenediamines **8a**–**e** with benzaldehydes **6**, **7**, **12** and **13** containing 1,4-disubstituted-1,2,3-triazoles prepared via a well-known Cu(I)-catalyzed azide-alkyne cycloaddition reaction.

The results of in vitro studies of antiproliferative activity against one non-tumor and seven cancer cell lines indicate that the introduction of a linker into bromine-substituted non-amidine compounds increases the selectivity and decreases the activity against the carcinoma cell lines tested (HeLa and CaCo2). Chlorine derivatives with bromine and without the linker have selective activity against all cell lines tested. All three non-amidine derivatives with bromine and with a linker showed selective activity against leukemia and lymphoma cells. Non-amidine compounds with a linker showed higher activity against leukemia and lymphoma cells after the introduction of bromine. These compounds affect different phases of the cell cycle, leading to changes in cell proliferation and growth. Cell cycle inhibition may be a key factor in the potential anti-cancer properties of these compounds.

A molecular docking study revealed that the quinoline–benzimidazole hybrid with propylamine at the C-5 position of the benzimidazole moiety, compound **14e**, had the highest affinity to inhibit TAO2 protein kinases, whose activity has been linked to DNA damage and cancer proliferation. This compound showed high and selective antiproliferative activity against lymphoma cell line. Despite its excellent drug-like properties, compound **14e** is not suitable for oral administration due to some deviations in its bioavailability.

Further research and investigation into the mechanisms of action and potential therapeutic applications of these hybrids would be necessary to fully understand their effects on the cell cycle and their ability to induce apoptosis as a mode of treated cell death.

## Figures and Tables

**Table 1:** Sensitivity of human tumor and normal cells to investigated compounds expressed as **IC_50_^a^** value.

		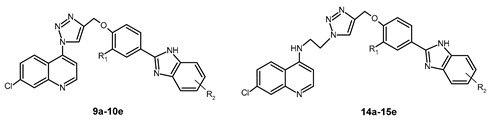						
Comp.	R_1_	R_2_	MRC-5	HeLa	CaCo-2	THP-1	Hut78	HL-60
**9a**	H	H	>100	>100	>100	>100	100	>100
**9b**	H	Cl	100	94.1	>100	>100	92.9 ± 4.3	>100
**9c**	H	OCH_3_	2.71	7.53	8.3	0.18	0.2	1.02
**9d**	H	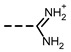	>100	>100	>100	>100	2.52	>100
**9e**	H	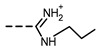	>100	>100	>100	>100	10.2	98.3
**10a**	Br	H	52.1	10.0	63.8	9.6	5.88	76.1
**10b**	Br	Cl	>100	8.11	10.9	10.6	6.43	55.2
**10c**	Br	OCH_3_	8.54	9.86	9.92	6.23	3.89	18.2
**10d**	Br	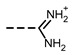	>100	>100	>100	>100	>100	>100
**10e**	Br	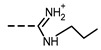	>100	>100	>100	>100	9.68	93.2
**14a**	H	H	>100	>100	>100	>100	>100	>100
**14b**	H	Cl	>100	>100	>100	>100	>100	>100
**14c**	H	OCH_3_	>100	>100	>100	7.31	>100	>100
**14d**	H	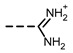	>100	>100	>100	>100	10.51	>100
**14e**	H	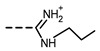	>100	>100	>100	>100	10.86	>100
**15a**	Br	H	>100	>100	>100	4.66	7.88	11.63
**15b**	Br	Cl	>100	>100	>100	2.06	4.91	1.24
**15c**	Br	OCH_3_	>100	>100	>100	1.65	3.98	2.62
**15d**	Br	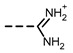	>100	>100	>100	>100	>100	>100
**15e**	Br	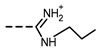	>100	>100	>100	>100	7.83	>100
**5-FU**			54.1	8.2	5.9	76.4	>100	>100

^a^ IC_50_—concentration of the compound that inhibits cell growth by 50%. Data represent mean IC_50_ (µM) values ± standard deviation (SD) from three independent experiments. Exponentially growing cells were treated with compounds during 72 h. Cytotoxicity was analyzed using MTT survival assay. **5-FU**: 5-fluorouracil.

**Table 2:** Calculated ADME, pharmacokinetic and druglike properties of the analyzed compounds.

Comp.	MW *	Csp3	RB	HBA	HBD	TPSA	XLOGP3	MLOGP	ESOL Log S	Water Sol.	GIA	BBBP	PgpS	LR
**9a**	452.9	0.04	5	5	1	81.51	4.97	3.49	−6.12	Poorly	High	No	Yes	0
**9b**	487.34	0.04	5	5	1	81.51	5.6	3.96	−6.71	Poorly	High	No	Yes	0
**9c**	482.92	0.08	6	6	1	90.74	4.94	3.17	−6.18	Poorly	High	No	Yes	0
**9d**	495.94	0.04	6	5	3	133.12	3.94	2.87	−5.62	Poorly	High	No	Yes	0
**9e**	538.02	0.14	9	5	3	119.13	5.23	3.45	−6.45	Poorly	High	No	Yes	1
**10a**	531.79	0.04	5	5	1	81.51	5.66	4.32	−7.03	Poorly	High	No	No	2
**10b**	566.24	0.04	5	5	1	81.51	6.29	4.79	−7.62	Poorly	Low	No	No	2
**10c**	561.82	0.08	6	6	1	90.74	5.63	3.73	−7.09	Poorly	Low	No	No	1
**10d**	574.84	0.04	6	5	3	133.12	4.63	3.43	−6.52	Poorly	Low	No	Yes	1
**10e**	616.92	0.14	9	5	3	119.13	5.92	4.01	−7.36	Poorly	Low	No	No	1
**14a**	495.96	0.11	8	5	2	93.54	4.93	3.1	−6.11	Poorly	High	No	Yes	0
**14b**	530.41	0.11	8	5	2	93.54	5.55	3.56	−6.7	Poorly	High	No	No	1
**14c**	525.99	0.14	9	6	2	102.77	4.9	2.78	−6.18	Poorly	High	No	Yes	1
**14d**	539.01	0.11	9	5	4	145.15	3.89	2.49	−5.61	Poorly	Low	No	Yes	1
**14e**	581.09	0.19	12	5	4	131.16	5.19	3.06	−6.45	Poorly	Low	No	No	1
**15a**	564.86	0.15	9	5	2	93.01	3.74	3.03	−5.52	Moderately	High	No	Yes	1
**15b**	599.31	0.15	9	5	2	93.01	4.37	3.49	−6.11	Poorly	High	No	No	1
**15c**	594.89	0.19	10	6	2	102.24	3.72	2.71	−5.6	Moderately	High	No	Yes	1
**15d**	607.91	0.15	10	5	4	144.62	2.71	2.69	−5.04	Moderately	Low	No	Yes	1
**15e**	649.99	0.23	13	5	4	130.63	2.71	2.69	−5.04	Moderately	Low	No	Yes	1

* MW (molecular weight, g/mol), Csp^3^ (fraction of carbons in the sp^3^ hybridization); RB (number of rotatable bonds); HBA (number of hydrogen-bond acceptors); HBD (number of hydrogen-bond donors); TPSA (topological polar surface area/Å^2^); XLOGP3 and MLOGP (lipophilicity descriptors); ESOL Log S (logarithm of the molar solubility in water); Water sol. (water solubility class according the Log S scale); GIA (gastrointestinal absorption); BBBP (blood–brain barrier permeation); PgpS (P-glycoprotein substrate); LR (number of Lipinski rule violations).

**Table 3:** Total binding energies (kcal/mol) of quinoline–benzimidazole hybrids and standard ligand staurosporine (STU) on TAO2 kinase domain (PDB: 2GCD) with percentage ratio of vdW (van der Waals); (Hbond) hydrogen-bonding; (Elec) electrostatic interactions (%).

Compound	Energy	VDW	HBond	Elec
**14e**	−140.44	79.20	20.80	0.00
**STU**	−137.99	89.85	10.15	0.00
**9e**	−131.00	79.16	20.84	0.00
**14d**	−127.85	80.96	19.04	0.00
**9d**	−127.67	72.83	24.98	2.19
**10e**	−124.68	86.23	13.77	0.00
**9c**	−123.88	84.30	15.70	0.00
**10c**	−123.41	78.59	21.41	0.00
**9a**	−122.31	79.50	20.50	0.00
**14a**	−120.23	83.55	16.45	0.00
**10d**	−119.16	81.20	16.97	1.83
**10b**	−118.41	74.77	25.23	0.00
**14c**	−116.35	83.43	16.57	0.00
**9b**	−115.47	85.73	14.27	0.00
**15e**	−114.44	84.73	15.27	0.00
**15c**	−111.56	87.59	12.41	0.00
**14b**	−110.44	81.86	18.14	0.00
**10a**	−106.61	83.64	16.36	0.00
**15a**	−105.91	88.73	11.27	0.00
**15d**	−105.48	88.01	11.99	0.00
**15b**	−101.95	72.16	27.84	0.00

**Table 4:** The energies (kcal/mol) of the main interactions between TAO2 kinase domain and compound **14e**.

H Bond	Energy	Van der Waals Interaction	Energy
M-Ile34	−2.30	M-Ile34	−3.95
M-His36	−3.50	M-Gly35	−7.69
S-Glu76	−6.90	M-His36	−7.87
M-Leu80	−1.60	S-His36	−1.21
M-Ile89	−3.50	S-Phe39	−3.82
S-Asp114	−3.50	S-Val42	−3.97
S-Glu117	−5.41	S-Lys57	−6.50
M-Asp169	−2.50	M-Leu80	−3.34
		S-Leu80	−11.43
		M-Ile89	−1.89
		M-Gln90	−7.64
		S-Met105	−8.39
		S-Asp114	−2.81
		S-Glu117	−3.38
		M-Gly168	−4.74
		M-Asp169	−3.88
		S-Asp169	−6.43
		S-Phe170	−3.39
		S-Met312	−2.46
		S-Lys314	−5.48

(M = main chain; S = side chain).

## Data Availability

Not applicable.

## Supplementary Materials

The following supporting information can be downloaded at: https://www.mdpi.com/article/10.3390/molecules28196950/s1. Table S1: Bioavailability radars for the 20 quinoline-benzimidazole hybrids. The pink area represents the optimal range for each property (lipophilicity (XLOGP3); size (MW); polarity (TPSA); water sol-ubility (log S); saturation (Fraction Csp3); and flexibility (number of rotatable bonds, FLEX).

## Sample Availability

Samples of the compounds are not available from the authors.
