# Supplementary material for: Novel 1,2,3-Triazole-Containing Quinoline–Benzimidazole Hybrids: Synthesis, Antiproliferative Activity, In Silico ADME Predictions, and Docking
Source: Molecules. 2023 Oct 6;28(19):6950. doi: 10.3390/molecules28196950 (PMC10574761; doi:10.3390/molecules28196950)
Supplement: Supplementary file 1 [file molecules-28-06950-s001.zip › molecules-2639926-supplementary.pdf]

Table S1. Bioavailability radars for the 20 quinoline-benzimidazole hybrids. The pink area represents the optimal range for each property (lipophilicity (XLOGP3); size (MW); polarity (TPSA); water sol-ubility (log S); saturation (Fraction Csp^3); and flexibility (number of rotatable bonds, FLEX).

			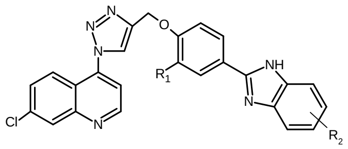 **9a-10e** 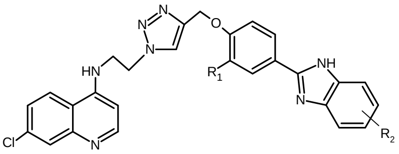 **14a-15e**
**Comp.**	**R1**	**R2**	
**9a**	H	H	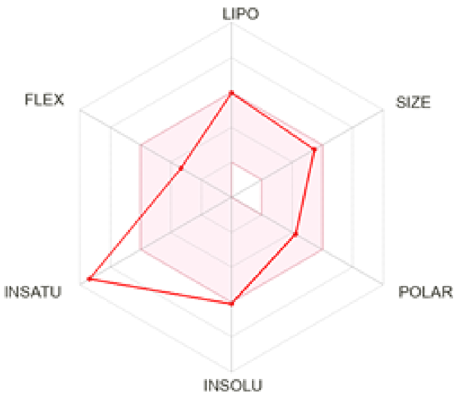
**9b**	H	Cl	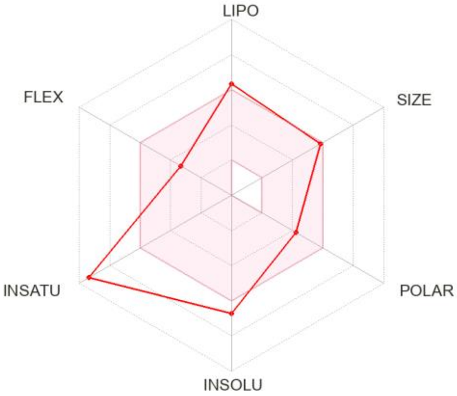

**9c**	H	OCH_3	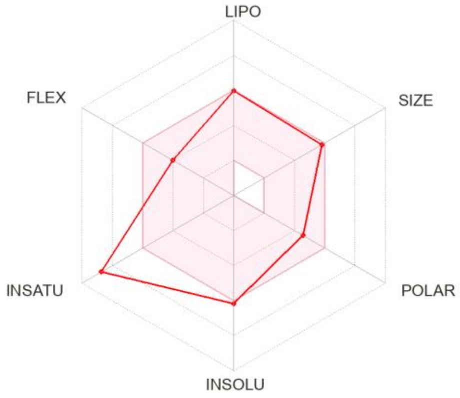
**9d**	H	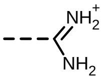	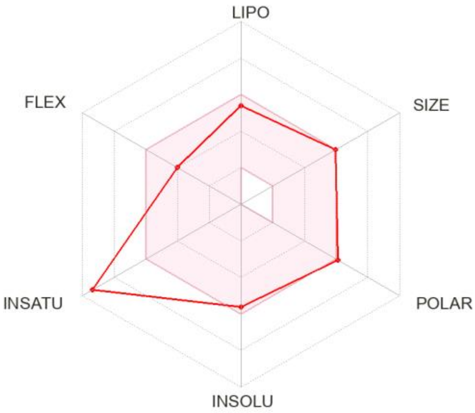
**9e**	H	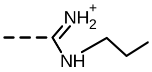	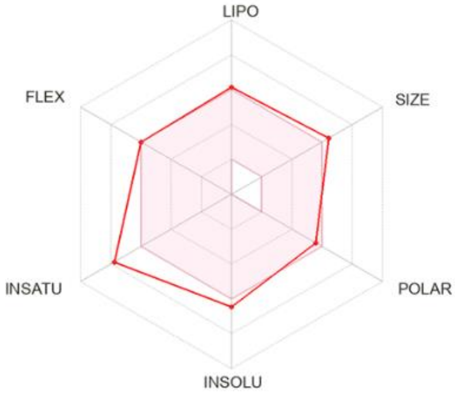

**10a**	Br	H	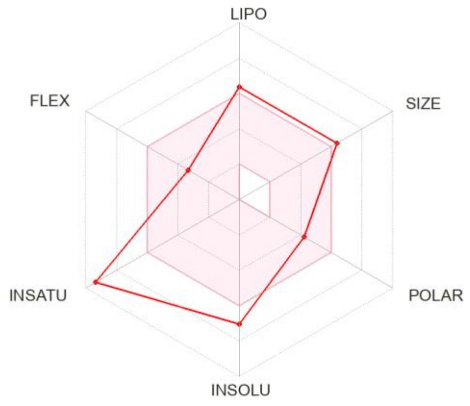
**10b**	Br	Cl	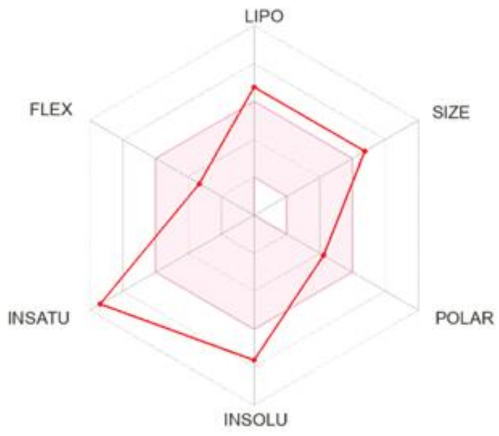
**10c**	Br	OCH_3	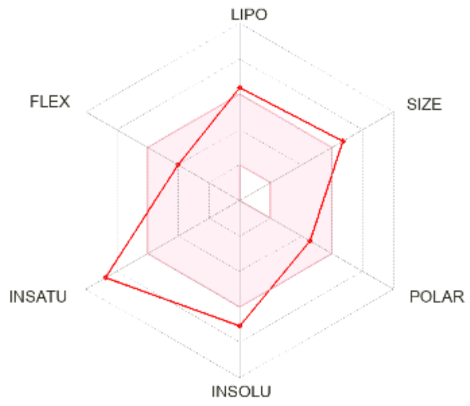

**10d**	Br	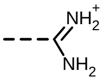	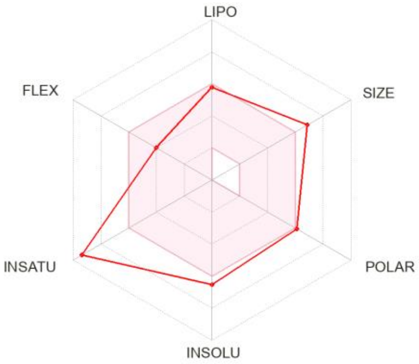
**10e**	Br	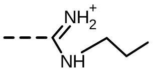	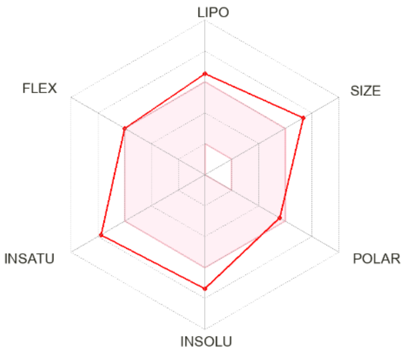
**14a**	H	H	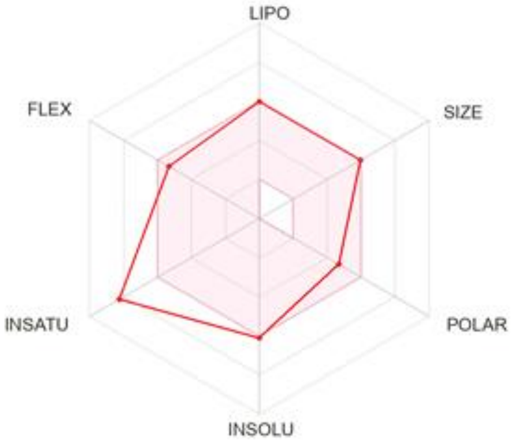

**14b**	H	Cl	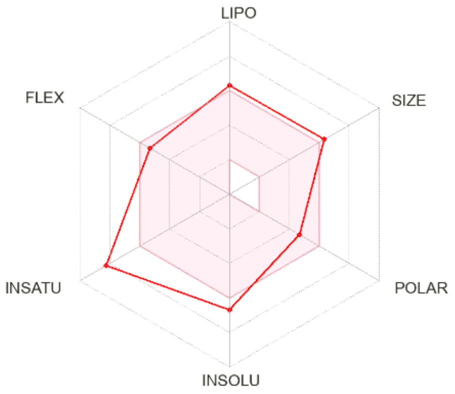
**14c**	H	OCH_3	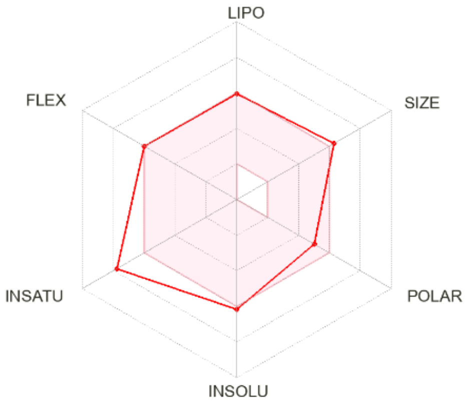
**14d**	H	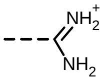	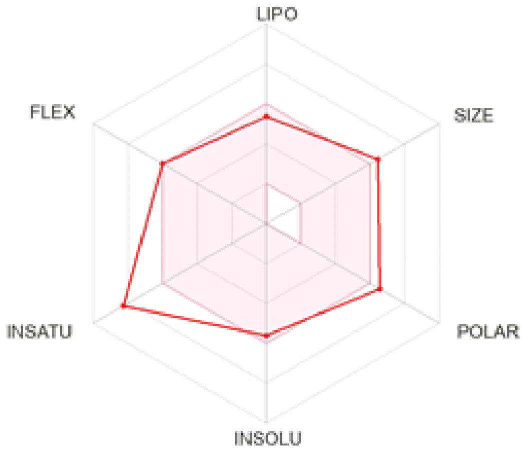

**14e**	H	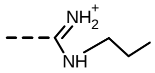	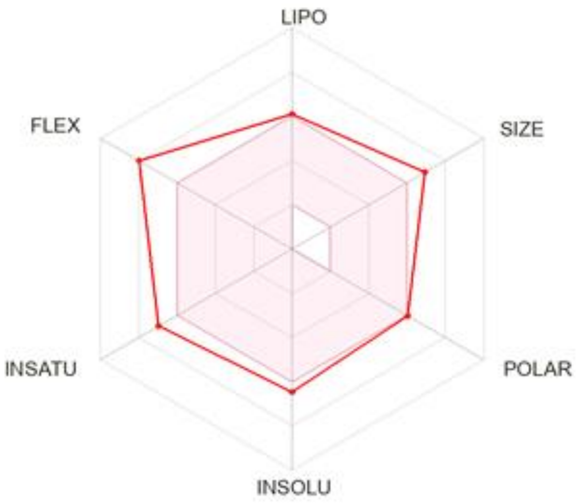
**15a**	Br	H	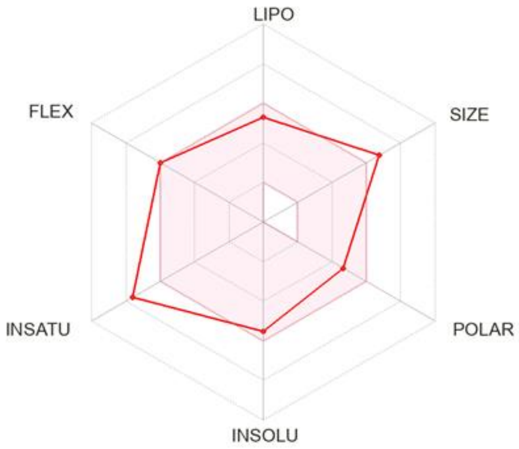
**15b**	Br	Cl	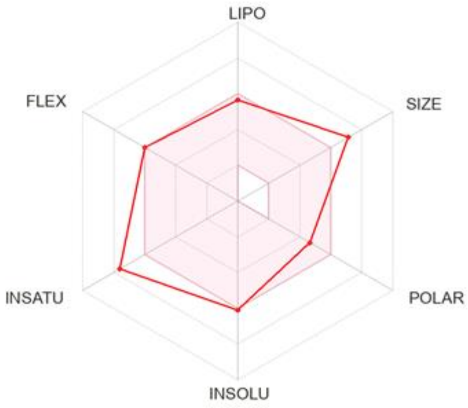

<table>
<tr><td>15c</td><td>Br</td><td>OCH_3</td><td>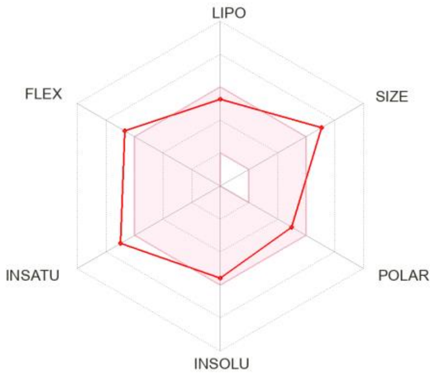
</td></tr>
<tr><td>15d</td><td>Br</td><td>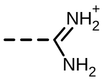
</td><td>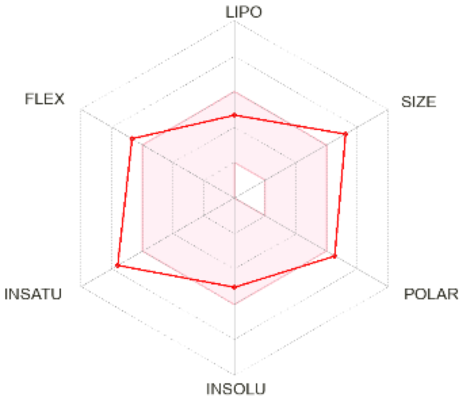
</td></tr>
<tr><td>15e</td><td>Br</td><td>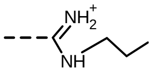
</td><td>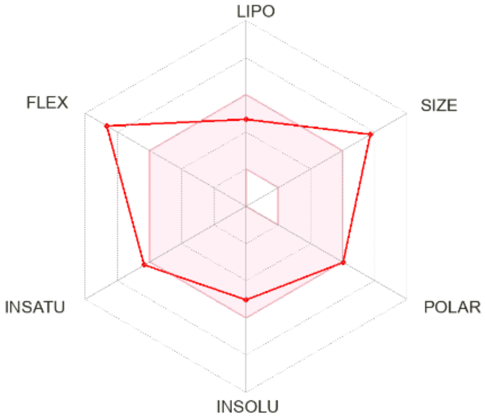
</td></tr>
</table>
